# Making the Complicated Simple: A Minimizing Carrier Strategy on Innovative Nanopesticides

**DOI:** 10.1007/s40820-024-01413-5

**Published:** 2024-05-14

**Authors:** Wenjie Shangguan, Qiliang Huang, Huiping Chen, Yingying Zheng, Pengyue Zhao, Chong Cao, Manli Yu, Yongsong Cao, Lidong Cao

**Affiliations:** 1 State Key Laboratory for Biology of Plant Diseases and Insect Pests , Institute of Plant Protection, Chinese Academy of Agricultural Sciences, Beijing, 100193 People’s Republic of China; 2 College of Plant Protection, China Agricultural University, Beijing, 100193 People’s Republic of China; 3 State Key Laboratory of Element-Organic Chemistry, Department of Chemical Biology, College of Chemistry, Nankai University, Tianjin, 300071 People’s Republic of China

**Keywords:** Nanopesticides, Green agrochemicals, Self-assembly, Nanomaterial safety, Sustainable development strategies

## Abstract

The flourishing progress in nanotechnology offers boundless opportunities for agriculture, particularly in the realm of nanopesticides research and development. However, concerns have been raised regarding the human and environmental safety issues stemming from the unrestrained use of non-therapeutic nanomaterials in nanopesticides. It is also important to consider whether the current development strategy of nanopesticides based on nanocarriers can strike a balance between investment and return, and if the complex material composition genuinely improves the efficiency, safety, and circularity of nanopesticides. Herein, we introduced the concept of nanopesticides with minimizing carriers (NMC) prepared through prodrug design and molecular self-assembly emerging as practical tools to address the current limitations, and compared it with nanopesticides employing non-therapeutic nanomaterials as carriers (NNC). We further summarized the current development strategy of NMC and examined potential challenges in its preparation, performance, and production. Overall, we asserted that the development of NMC systems can serve as the innovative driving force catalyzing a green and efficient revolution in nanopesticides, offering a way out of the current predicament.

**Supplementary Information:**

The online version contains supplementary material available at 10.1007/s40820-024-01413-5.

## Highlights

- Nanopesticides with minimizing carrier were prepared through prodrug molecular design and molecular self-assembly.
- Nanopesticides with minimizing carrier are expected to solve the environmental risks caused by the unrestricted introduction of nanomaterials.
- Future development and challenges of nanopesticides with minimizing carrier.

**Supplementary Information:**

The online version contains supplementary material available at 10.1007/s40820-024-01413-5.

## Graphical abstract

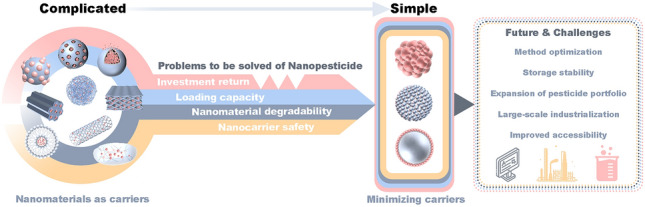

## Introduction

In the contemporary era, transformative changes are needed in agricultural and food systems to cope with the impacts of global climate change [1]. Pesticides are widely used as a vital component of this system, but the escalating issue of resistance conceals a substantial crisis [2]. Concerns regarding toxicity, pollution, and oversight have arisen successively [3]. Nowadays, science-driven advances form the cornerstone for addressing these challenges [4], with nanotechnology emerging as a promising frontier for revolutionizing pesticides [5]. Nanopesticides have emerged as a development with significant potential, and their importance and priority have been widely recognized [6]. In this field, using nanomaterials to load active ingredients stands out as a pivotal strategy for constructing nanoscale delivery platforms, which currently representing the most mainstream research and development concept. Compared with traditional pesticides, nanopesticides can be endowed with stimulus-responsive functions through specific materials and can be efficiently delivered to targets through small size effects [7, 8]. Nanopesticides play a pivotal role in enhancing food security and fostering sustainable practices in crop protection [9–11].

**Fig. 1:**
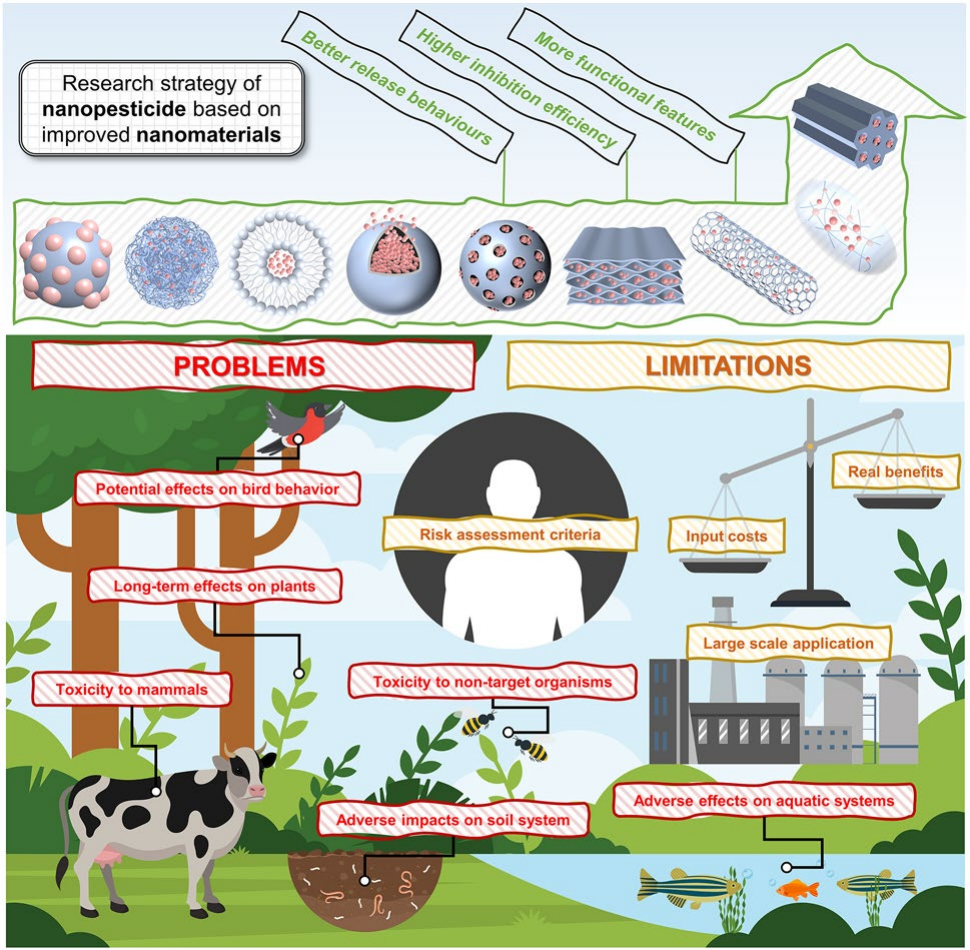
Advantages and challenges of utilizing nanomaterials in nanopesticides

As mentioned above, research strategy of nanopesticides is predominantly based on developing diverse nanomaterials as carriers to provide better release behavior, higher inhibition efficiency, and more functional features for this delivery system (Fig. 1). However, has the overall efficiency of current nanopesticides truly achieved a significant leap from millimeters or micrometers scales to nanometer scales? The size effect of nanopesticides is influenced by multiple factors, and arriving at more accurate conclusions necessitates prolonged and thorough discussions. Regrettably, existing research data does not appear to demonstrate that the enhancement of nanopesticides has achieved the most optimal results [12]. Simultaneously, it is crucial to emphasize that the development of pesticide products is highly dependent on cost considerations. In the present progression of nanopesticides, the excessive modification of nanomaterials within nanoscale delivery platforms through intricate processes often diverges from the core principles of sustainable development. This deviation is notable as sustainable development underscores efficiency, safety, and circularity, which are important guiding principles for nanopesticides research [13, 14].

Given the background outlined above, the next crucial topic for in-depth discussion is the safety of current nanopesticide research strategies centered around nanomaterials. Compared with medicine, the process of pesticide delivery and application scenario are open and complex, involving the entire ecosystem, and its potential process losses and residual hazards receive widespread attention. Before nanopesticide products enter the market, the establishment of a comprehensive framework for risk assessment is imperative to ensure compliance with the demands of sustainable intensification in the future. On the one hand, the large-scale application of engineered nanomaterials (EEMs) has indeed promoted the development of nanopesticides, but, on the other hand, addressing the inherent risks and limitations of the unrestrained introduction and proliferation of nanomaterials will be a challenge [15–17]. Acknowledgment is necessary for the potential long-term adverse effects of EEMs on agroecosystems [18], encompassing spanning soil, plants, and various organisms, including humans [19–22] (Fig. 1). The introduction of EEMs may also cause indirectly the harmful of nanoplastics on biological systems, which posing a significant threat to agricultural sustainability and food safety [23]. Furthermore, existing analyses indicate that a stringent screening process for certain EEMs is necessary to ensure that they meet the conditions for maximizing the net realized benefits in a specific agricultural application [24]. Collectively, the balance between input costs (material, energy input, usage amount, and labor and time costs) and realized benefits (crop yield, germination rate, and disease incidence) of EEMs, the effectiveness of risk assessment system, and the prospects of large-scale application are still controversial. Indeed, these issues have started to impede the progress of nanopesticides.

How to advance the development and application of nanopesticides in the future while reducing dependence on nanomaterials is an urgent issue deserving serious consideration. It is noteworthy to emphasize the growing importance of carrier-free nanodrugs in the medical field [25], which providing a reference for getting out of the current predicament [26, 27]. Carrier-free nanodrugs developed through prodrug preparation and molecular self-assembly exhibit exceptionally high loading capacity [28]. This system enhances overall safety through reducing non-therapeutic materials and increasing effective content. The advent of cost-effective and high-throughput self-assembly technologies has increased the scalability of this strategy [29]. Indeed, carrier-free is an ideal drug deliver platform. These approaches of minimizing carriers are pivotal in addressing the limitations of current nanoscale delivery platforms, particularly concerning aspects such as loading capacity and toxicity [30, 31]. Against this backdrop, strategies aimed at minimizing carriers are expected to be employed in the development of nanopesticides and offer potential solutions to current agriculture challenges. In recent years, some agricultural chemists have put forth substantial technological methods aimed at minimizing non-therapeutic carriers in nanopesticides. However, there is a notable lack of a systematic overview of this developmental direction, which is essential to provide comprehensive guidance for the creation of the new generation of nanopesticides.

In this review, we first summarize the development and characteristics of nanopesticides with minimizing carriers (NMC) prepared through prodrug design and molecular self-assembly, and compare them with nanopesticides employing non-therapeutic nanomaterials as carriers (NNC). Next, the development strategies and preparation mechanisms of recent research on NMC are analyzed, including reactions between small molecules, reactions between host–guest compounds and small molecules, and reactions between low molecular weight polymers and small molecules. Finally, challenges and opportunities for the future development of NMC are discussed based on current scientific research progress. We hope that this review effectively elucidates the significance of NMC development for nanopesticides and offers innovative ideas for the long-term green revolution of pesticide in the future.

## Progress and Properties

**Fig. 2:**
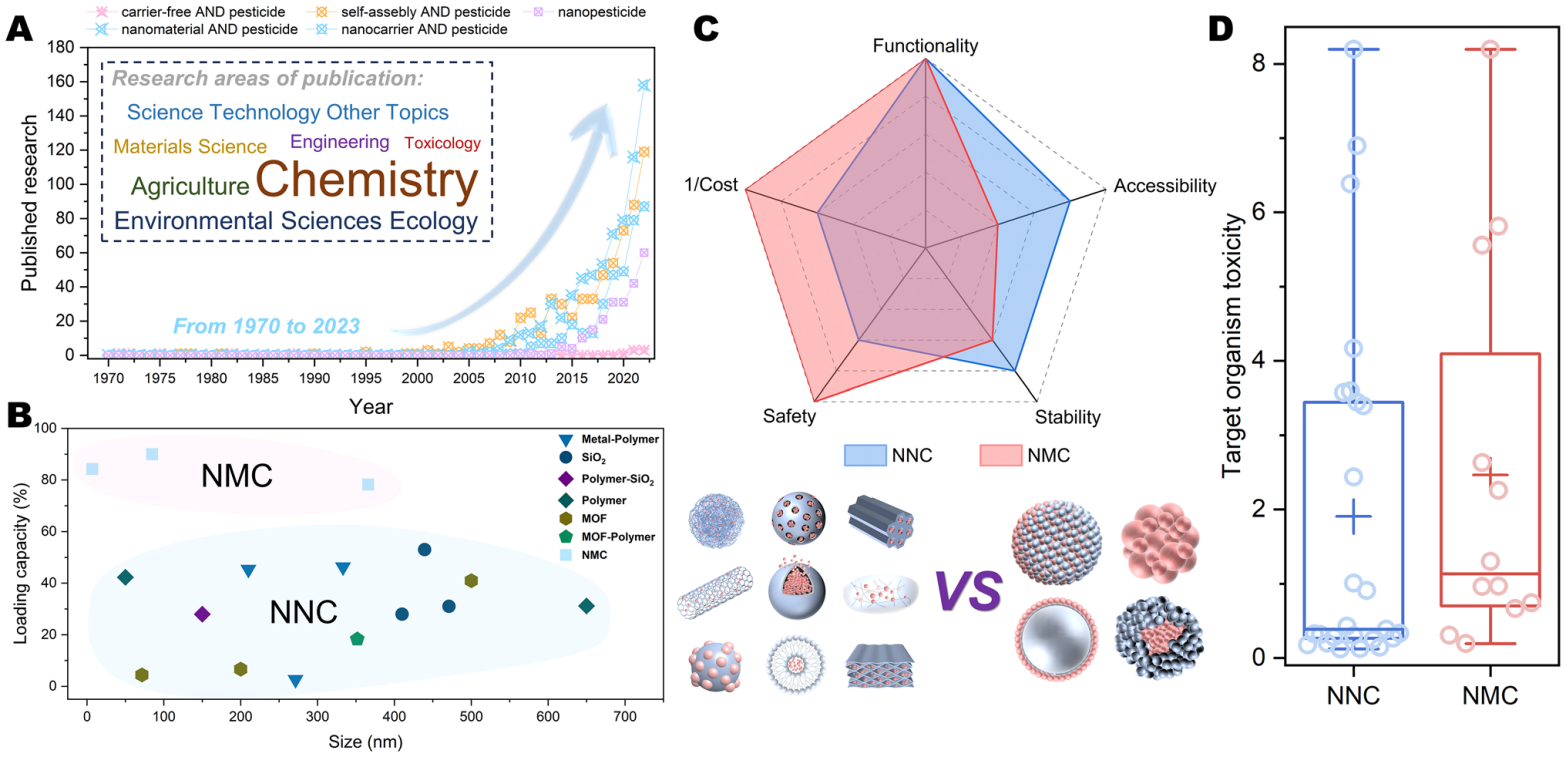
Development overview and comparison of properties of NMC and NNC. **A** Publication trends and research areas of NMC and NNC. Keyword search and paper collection source Web of Science, including only research papers. **B** A comparison of the loading capacity of NMC and NNC. Relevant information of cited references is in Table S1 of Supplementary Information. **C** A comparison of comprehensive properties of NMC and NNC. The evaluation criteria for the comprehensive properties of NMC and NNC based on the relevant data in Table S1-2 of Supplementary Information. **D** The target organism toxicity of NMC and NNC. The lower and upper ends of the box represent the 25th and 75th percentiles, respectively, while the line and cross inside the box denote the median and mean, respectively. The whiskers indicate the 90th and 10th percentiles. These parameters are derived from dose–response relationships, such as EC_50_ or LC_50_, which are inversely related to toxicity. Their inverse ratios are compared in the analysis. Relevant information of cited references is in Table S2-3 of Supplementary Information

As materials science and nanotechnology become integral to pesticide research, the past decade has witnessed rapid advancements in the realm of nanopesticides. In light of the observed trend in the number of published papers and the convergence of research fields, it can be anticipated that future investigations into nanopesticides will continue to thrive (Fig. 2A). The emergence of the NNC system primarily stems from the utilization of nanotechnology in pesticide formulations, and the carrier role played by nanoparticles in pesticides. In contrast with conventional pesticide formulations plagued by issues such as high organic solvent content, poor dispersibility, and short duration of action, the NNC system has advantages in enhancing the physical and chemical properties, achieving targeted and sustained release, and multifunctionality [32]. Therefore, the related patented products of NNC systems have been successfully introduced to the market. Examples include products such as Nualgi Foliar Spray, Dedalo Elite, and AZteroid FC 3.3 [9]. In addition, the expanding production scale of EEMs aligns with the feasibility of large-scale application of NNC systems in the future [24]. Through data retrieval, it can be seen that most of research articles on nanopesticides are driven by the functional modification of nanocarriers or the composite use of multiple nanomaterials. The delivery of genetic material via nanomaterials has also started to garner attention in the realms of crop engineering and plant protection [33, 34]. But researches on carrier-free nanopesticides are sparse. However, what cannot be ignored is the extensive research on molecular self-assembly and pesticide delivery during the development of nanopesticides [35, 36]. Molecular self-assembly makes the transformation from molecular entities to micron- or nanoscale substances [37], which is also one of the key foundations of current nanomedicine for active molecules delivery [38]. NMC, as a nanopesticide, relies on minimal or negligible use of nanomaterials to form nanoparticles, microspheres, vesicles, or other delivery systems (the molecular weight of all compounds cited in this article does not exceed 10 k). Molecular self-assembly stands out as one of the pivotal steps for obtaining this system. Therefore, molecular self-assembly, which have gained significant ground in research of pesticide delivery, has the potential to contribute to the continued development of NMC.

NMC typically boasts high loading capacity and mitigates the risk of toxic events associated with excessive use of nanomaterials for loading enhancement [30]. Figure 2B summarizes the loading capacity reported in recent studies of nanopesticides. The results indicated that, at comparable sizes, NMC exhibited significantly superior pesticide loading capacity compared to NNC, which encompassed SiO_2_, polymer, MOF, and their composites (Table S1). NNC employs these materials for pesticide loading through encapsulation, adsorption, or modification. In the NMC system, besides utilizing small molecule compounds, low molecular weight polymer can also be selected as one of the monomers participating in prodrug design and molecular self-assembly. For instance, polyethylenimine (PEI) molecules can be involved in the preparation of NMC system and can also serve as modified carriers in NNC system [39]. It is evident that the variances in preparation strategy and material choices between NMC and NNC systems directly result in essential difference in loading capacity between the two systems. For nanopesticides, the superior loading capacity exhibited by NMC delivery systems can play a crucial role in reducing the dose administered, offering a potential strategy to mitigate the development of pesticide resistance and improve safety [40].

When comparing the comprehensive performance of NNC and NMC (Fig. 2C), toxicity and safety need to be mentioned first. In fact, the standout characteristic of nanopesticides is that nanonization strengthens biological effects, such as biological barrier penetration, adjustment of reactive oxygen species (ROS), and modulation of plant metabolism and signaling pathways [41]. In the analysis conducted by Kah et al. in 2018 [13], the toxicity of nanopesticides to target organisms increased by 24% compared to non-nanopesticides. Similarly, in the study by Wang et al. in 2022 [12], this increase was reported to be 31.5%. These analyses, focusing on EEMs and NNC as the primary research subject, have revealed an augmentation in the toxic effects of nanopesticides. However, additional multidimensional assessment criteria may be necessary for evaluating toxicity to non-target organisms, particularly concerning genotoxicity against refractory substances. At the same time, attention should be given to controversial experimental results regarding the safety assessment of aquatic life [42]. The very direct introduction of nanomaterials that are difficult to degrade naturally into organisms and ecosystems could be a double-edged sword. This is likely to be one of the primary constraints on market regulation of NNC-based nanopesticides in the future [43]. Herein, we also conducted a preliminary comparison of the target organism toxicity of NMC and NNC (Fig. 2D and Table S2). The results indicate that the average value of target organism toxicity of NMC was 1.29 times that of NNC, suggesting that NMC may potentially offer efficacy advantages (Table S3). In terms of non-target organism toxicity, the water-only NMC system prepared by An et al. [44] showed low toxicity to non-target plant seeds, human epithelial cells, zebrafish, earthworms, and *Escherichia coli*. Furthermore, the presence of prodrugs may also enhance biosafety. These results indicate that pure NMC systems have the potential to mitigate the potential toxicity associated with organic solvents, surfactants, or nanomaterials. But due to the small sample size and the variability in biological test conditions, further studies are required for validation.

The functionality of nanopesticide systems currently includes basic loading and release functions as well as additional functions, such as nutritional functions and stress alleviation. The development of these functions mostly comes from the addition of exogenous compounds, so both NMC and NNC are easy to implement. Importantly, the stimulus-responsive capability of NMC can also be attributed to the reversibility of the structures formed through self-assembly, enabling NMC to response to environmental factors such as temperature and pH [45, 46]. Another potential advantage of NMC is cost-effectiveness, mainly attributed to exclude large-scale manufacturing of carriers and the improvement of efficacy. However, the absence of a protective carrier leads to limited stability for NMC, potentially hindering its delivery in complex agricultural environments. Additionally, another drawback of NMC is accessibility. Its preparation requires careful consideration of the physical and chemical properties of the reacted monomer molecules, as well as the properties of the resulting combined prodrug molecules, rendering it more challenging than the NNC system. The formation of NMC necessitates the design of specific molecular structures, and detailed adjustments to reaction conditions are also required. Therefore, the next chapter summarizes current preparation strategies of NMC and analyzes design concepts for future reference.

## Preparation Strategies

The design methodology of carrier-free nanodrugs in medicine can provide insightful references for NMC [28]. However, differences in application environments introduce variations in overall design considerations. While the concept of NMC is relatively new in the development of nanopesticides, several promising results have been reported. The studies primarily both innovated existing pesticide molecules through structural design to align with the conditions required for molecular self-assembly. For agricultural application, the preparation strategy of NMC is designed based on pesticide molecules, including between small molecules, between host–guest compounds and small molecules, and between low molecular polymers and small molecules. The resulting NMC exhibited response functions based on the specific target and the application scenario of the pesticide [47]. Additionally, enhancing the efficiency of pesticide application and minimizing the environmental toxicity of potent pesticides are also key considerations in the current design concepts for NMC [48].

### NMC Formed Between Small Molecules

The molecular self-assembly is a bottom-up process, necessitating the aggregation of free small molecules for the formation of the nano-delivery system. However, it is difficult to rely solely on an ordinary pesticide molecule in establishing relatively stable nano-assembly systems. Therefore, it becomes necessary to construct prodrug conjugates with amphipathic properties. In addition to serving as the foundation for the formation of macroscopic nanoparticles, these prodrug conjugates typically exhibit more optimized physical and chemical properties [49, 50]. For pesticide application, the specific objectives include achieving higher efficacy, minimizing environmental toxicity, and incorporating specific response functions. The following are the specific strategies for constructing prodrug conjugates through reactions between small molecules.

**Fig. 3:**
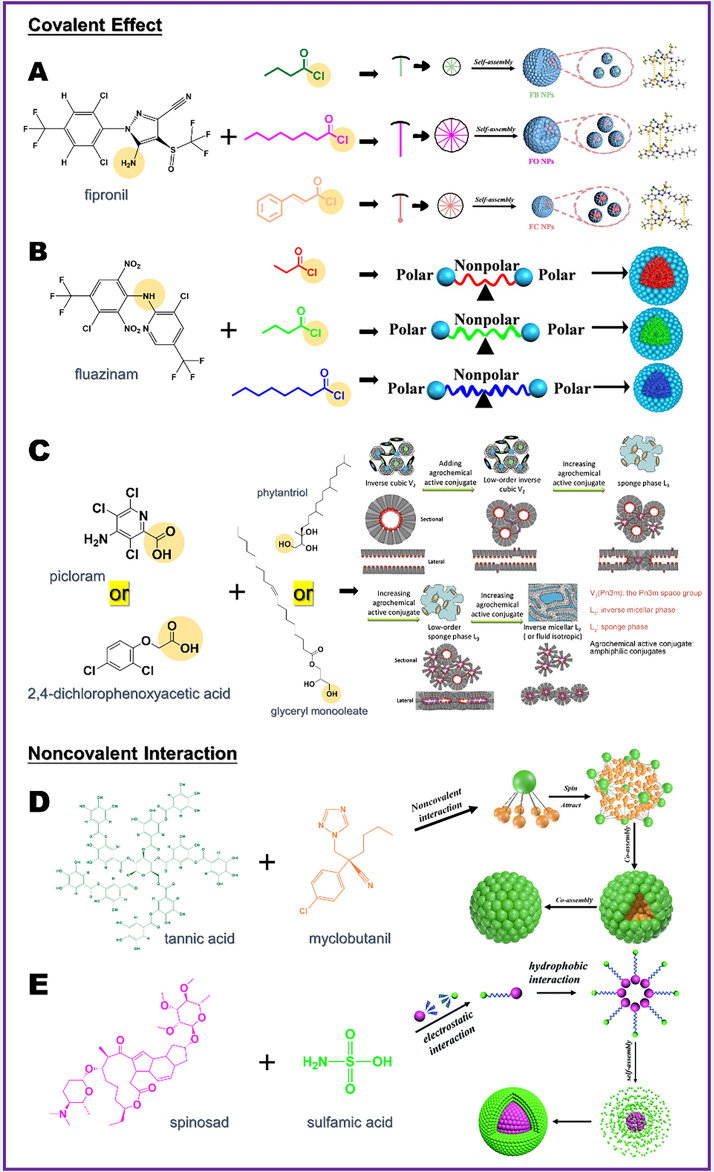
NMC preparation strategy based on the interaction between small molecules. A Schematic illustration of fipronil and three acyl chlorides forming NMC based on amide bond [52], copyright 2022, Elsevier. B Schematic illustration of fluazinam and three acyl chlorides forming NMC based on amide bond [47], copyright 2023, American Chemical Society. C Schematic illustration of 2,4-dichlorophenoxyacetic acid or picloraml and phytantriol or glyceryl monooleate forming NMC based on ester bond [54], copyright 2018, Elsevier. D Schematic illustration of myclobutanil and tannic acid forming NMC based on noncovalent interaction [40], copyright 2023, Wiley Online. E Schematic illustration of spinosad and sulfamic acid forming NMC based on noncovalent interaction [58], copyright 2021, Royal Society of Chemistry

i) Some pesticide molecules with amino group can be bonded to small molecules, enabling the construction of prodrug conjugates [51]. Tian et al. [52] conjugated fipronil with three natural carboxylic acids of varying polarities, strategically modulating the intramolecular polar balancing forces of the conjugate to achieve nanospheres with optimal physical and chemical properties (Fig. 3A). Simultaneously, the resulting amphipathic molecules enhanced affinity with the leaf surface, thereby reducing the surface tension of pesticide droplets and enhancing deposition efficiency. Additionally, the toxicity of zebrafish exposed to molecules incorporating long carbon chains and aromatic rings was reduced by nearly four times compared to fipronil. Also, utilizing the amino group on fipronil, Zhao et al. [53] introduced 2-sulfobenzaldehyde and facilitated its combination to create an imine covalent bond, subsequently self-assembling into nanomicelles with a diameter of approximately 16.9 nm. The dynamic nature of the imine construction enables the system to precisely release highly active fipronil under conditions of high humidity and weak alkalinity. Utilizing the amide bond-forming reaction, Zhang et al. [47] conjugated fluazinam with alkyl aliphatic acids of varying lengths (Fig. 3B). The results indicated an inverse relationship between the degradation rate of the amide bond in papain and the length of the conjugated alkyl fatty acid, allowing for the customization of the enzyme-responsive NMC.
ii) Esterification reactions can be employed to generate amphiphilic alkylated prodrug conjugates. Research by Liu et al. [54] showed that 2,4-dichlorophenoxyacetic acid or picloram can self-assemble in water to form nanoparticles with a liquid crystal structure after conjugation with phytantriol or glyceryl monooleate (Fig. 3C). This nanoparticle with a liquid crystal structure is a promising pesticide delivery system in terms of its surfactant reduction for improved environmental safety [55]. In comparison with simple alkyl ester conjugates, amphiphilic conjugates reduced the loss of inverse cubic structure, addressing the issue of low pesticide loading in the original liquid crystal system. Enhancements in ester systems will contribute to the future advancement of nanopesticides featuring liquid crystal structures.
iii) Two small molecules with specific structures can be constructed into self-assembling nanoparticles through noncovalent interactions. Tian et al. [48] employed berberine and curcumin, both possessing bactericidal effects extracted from medicinal plants, to undergo supramolecular self-assembly through various noncovalent interactions. This study introduces innovative approaches for creating NMC systems incorporating botanical pesticides. Based on nanoprecipitation method, Li et al. [40] regulated the solvent for precipitating myclobutanil, and during this precipitation process, tannic acid, possessing a noncovalent molecular structure, could interact with myclobutanil. The bound molecules persistently rotated and intertwined through noncovalent interactions, culminating in the formation of a spherical three-dimensional structure (Fig. 3D). Tian et al. utilized the protonation of the tertiary amine group in spinosad to bind with sulfamic acid through noncovalent electrostatic interactions, subsequently undergoing winding and rotation to form a sphere (Fig. 3E). Among them, temperature, pH, molar ratio, and ionic strength might have an impact on co-assembly of this system. In the research conducted by Cui et al. [56], nanoparticles were produced using emamectin benzoate and sodium lignosulfonate through electrostatic self-assembly. The research results indicate that the physical and chemical properties of NMC system can be optimized through the adjustment of surfactants.

In addition to the aforementioned preparation concepts, the co-crystallization strategy, rooted in the supramolecular self-assembly mechanism, is also a viable approach for developing NMC [57]. Significantly, NMC constructed with two pesticide molecules often exhibit synergistic effects, which requires further discussion in the future.

### NMC Formed Between Host–Guest Compounds and Small Molecules

The development of NMC based on prodrug conjugates via host–guest interactions stands out as one of the currently effective preparation strategies. Water-soluble host molecules employ the hydrophobic effect of the cavity to facilitate host–guest self-assembly with pesticide molecules [59]. Commonly utilized host molecules encompass pillar[*n*]arenes, cucurbit[*n*]urils, and cyclodextrins.

**Fig. 4:**
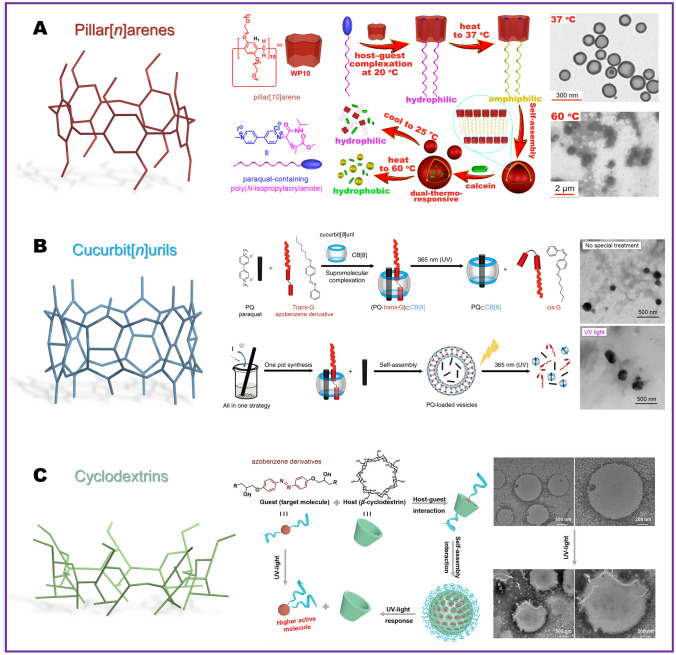
NMC preparation strategy based on the interaction between host–guest compounds and small molecules. **A** Schematic illustration of supramolecular complexes formed by host–guest interactions based on pillar[*10*]arenes [46], copyright 2016, American Chemical Society. **B** Schematic illustration of supramolecular complexes formed by host–guest interactions based on cucurbit[*8*]uril [63], copyright 2018, Springer Nature. **C** Schematic illustration of supramolecular complexes formed by host–guest interactions based on *β*-cyclodextrin [64], copyright 2023, Wiley Online

Designing supramolecular polymers for the highly toxic pesticide paraquat holds great significance in enhancing safety and functionality [60]. In Fig. 4A, based on the supramolecular chemistry of pillar[*n*]arenes, Chi et al. [46] presented a preparation method for NMC with dual-thermoresponsiveness. In their study, pillar[*10*]arene and paraquat derivatives formed a 1:2 [3] pseudorotaxane, which can self-assemble into vesicles in water above the lower critical solution temperature (LCST) of paraquat derivatives. Leveraging the different LCST behaviors of pillar[*10*]arene and poly(N-isopropylacrylamide) (the block where paraquat was introduced), dual-thermoresponsiveness was attained. Later, Song et al. [61] utilized pillar[*5*]arenes and benquitrione through selective dynamic self-assembly to create vesicles, which improved the spreading of pesticide droplets on the hydrophobic interface through host–guest and hydrogen bonding interactions.

Cucurbit[*n*]urils are also macrocyclic molecules capable of encapsulating many guest molecules [62], and the tunable stimulus responsiveness of the resulting complexes has garnered widespread attention. To also enhance the safety of paraquat, Gao et al. [63] devised supramolecular vesicles through molecular self-assembly relying on cucurbit[*8*]uril. In Fig. 4B, the researchers initially synthesized a hydrophobic azobenzene derivative (*Trans*-G), then created an amphiphilic ternary complex involving paraquat, *Trans*-G, and cucurbit[*8*]uril in water. Finally, the complex formed hollow spheres through self-assembly, which also capable of loading active substances, and achieved responsive release under ultraviolet light through the *trans*-to-*cis* isomerization of *Trans*-G. In a recent study by Ji et al. [45], highly bioactive carbazole-modified amphiphilic quaternary ammonium salts with cationic *N*-benzylimidazolium pendants were prepared. Then, the host cucurbit[*7*]uril was introduced to construct a supramolecular system, regulating its release by the addition of competitive molecules (such as 1-adamantanamine hydrochloride).

Cyclodextrins, especially *β*-cyclodextrin, are recognized as ideal macrocyclic host molecules in the agricultural field due to their good physical and chemical properties and cost-effectiveness. In Yang et al.’s study [64], azobenzene analogs featuring the pharmacophore-isopropanolamine moiety were designed as guest molecules in *β*-cyclodextrin due to their photoresponsive property and antibacterial function (Fig. 4C). This light-responsive antibacterial system formed through supramolecular self-assembly, which can inhibit up to 55.84% against rice bacterial blight pathogens by UV–Vis exposure. Importantly, it has been demonstrated to be non-toxic to various non-target organisms (plant, aquatic organism, and human cell lines). Also based on the host–guest interaction of *β*-cyclodextrin, Ji et al. [65] synthesized adamantane-decorated 1,3,4-oxadiazoles to enhance the formation of antibacterial supramolecular complexes. After the host–guest assembly was formed, the water solubility and foliar surface wettability were significantly improved and improved the control effect against rice bacterial blight and kiwi canker diseases.

### NMC Formed Between Low Molecular Weight Polymers and Small Molecules

The utilization of low molecular weight polymers and small molecules to create nanoassemblies is a distinctive strategy for NMC preparation, which is different from the large use of high polymers simply as carriers. Low molecular weight polymers lack molecular entanglement and have shorter chain segments, leading to faster degradation in nature. Higher molecular mobility facilitates participation in the molecular self-assembly of the solution system. Conjugating small molecules to these polymers alters the water solubility, safety, and dispersion of NMC while maintaining a higher loading capacity [28].

**Fig. 5:**
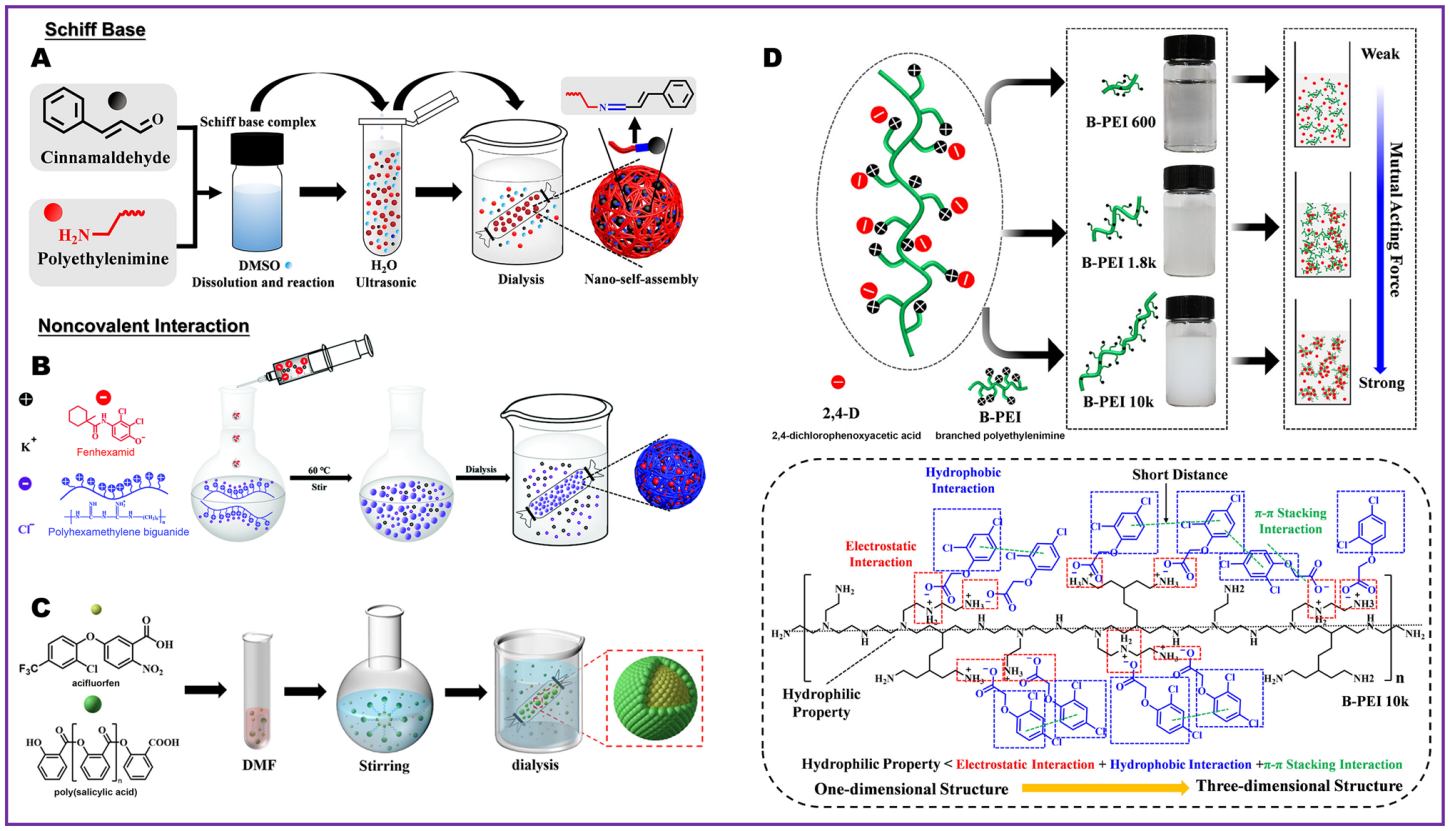
NMC preparation strategy based on the interaction between low molecular weight polymers and small molecules. **A** Schematic illustration of supramolecular complexes formed by PEI and cinnamaldehyde based on Schiff base [66], copyright 2023, Elsevier. **B** Schematic illustration of supramolecular complexes formed by polyhexamethylene biguanide and fenhexamid based on noncovalent interaction [67], copyright 2021, Royal Society of Chemistry. **C** Schematic illustration of supramolecular complexes formed by poly(salicylic acid) and acifluorfen based on noncovalent interaction [68], copyright 2023, American Chemical Society. **D** Schematic illustration of possible acting forces driving the formation of the self-assembly between low molecular weight polymers and small molecules [69], copyright 2022, American Chemical Society

In the previous studies, Tang et al. [66] employed cinnamaldehyde and branched PEI of varying molecular weights to create Schiff base complexes, which subsequently self-assembled into nanoparticles through noncovalent interactions (Fig. 5A). The release of this NMC with Schiff base intelligently responds to the acidic biological microenvironment created by some fungal pathogens during infection. In another study by Tang et al. [67], it was observed that cycloheximide and polyhexamethylene biguanide self-assemble in aqueous solution to form nanospheres (Fig. 5B). The process of self-assembly is governed by the electrostatic attraction between the anions of fenhexamid and the skeleton of polyhexamethylene biguanide, as well as the hydrophobic interaction force induced by the anions of fenhexamid. In Fig. 5C, Wang et al. [68] synthesized amphiphilic complexes capable of self-assembly into nanoparticles in water. Subsequently, poly(salicylic acid) attracted acifluorfen to interact with it through noncovalent interactions, including hydrogen bonding and *π*−*π* stacking, ultimately resulting in the formation of co-assembled nanoparticles. The results indicated that stable co-assemblies tend to form when there was a balance between attractive and electrostatic repulsive forces between polymers and small molecules.

In the co-assembled between polymers and small molecules to form NMC, the average molecular weight of the polymer often plays a crucial role. For example, branched PEI with a lower average molecular weight cannot form a strong interaction with 2,4-dichlorophenoxyacetic acid. As the average molecular weight increases, its longer main chain exhibited various forces that drive it to entangle with each other, forming a three-dimensional structure (Fig. 5D). However, this does not imply that infinitely increasing the length of the main chain will form better co-assembly. Excessively long main chains may hinder rotational winding. When designing future NMC systems, the suitable average molecular weight of the selected polymer can be further determined by analyzing the appearance, pH value, and conductivity changes of the reactants [69]. In addition, the development of supramolecular biopolymers can also expand ideas for the preparation of this type of NMC [11].

## Conclusions and Perspectives

**Fig. 6:**
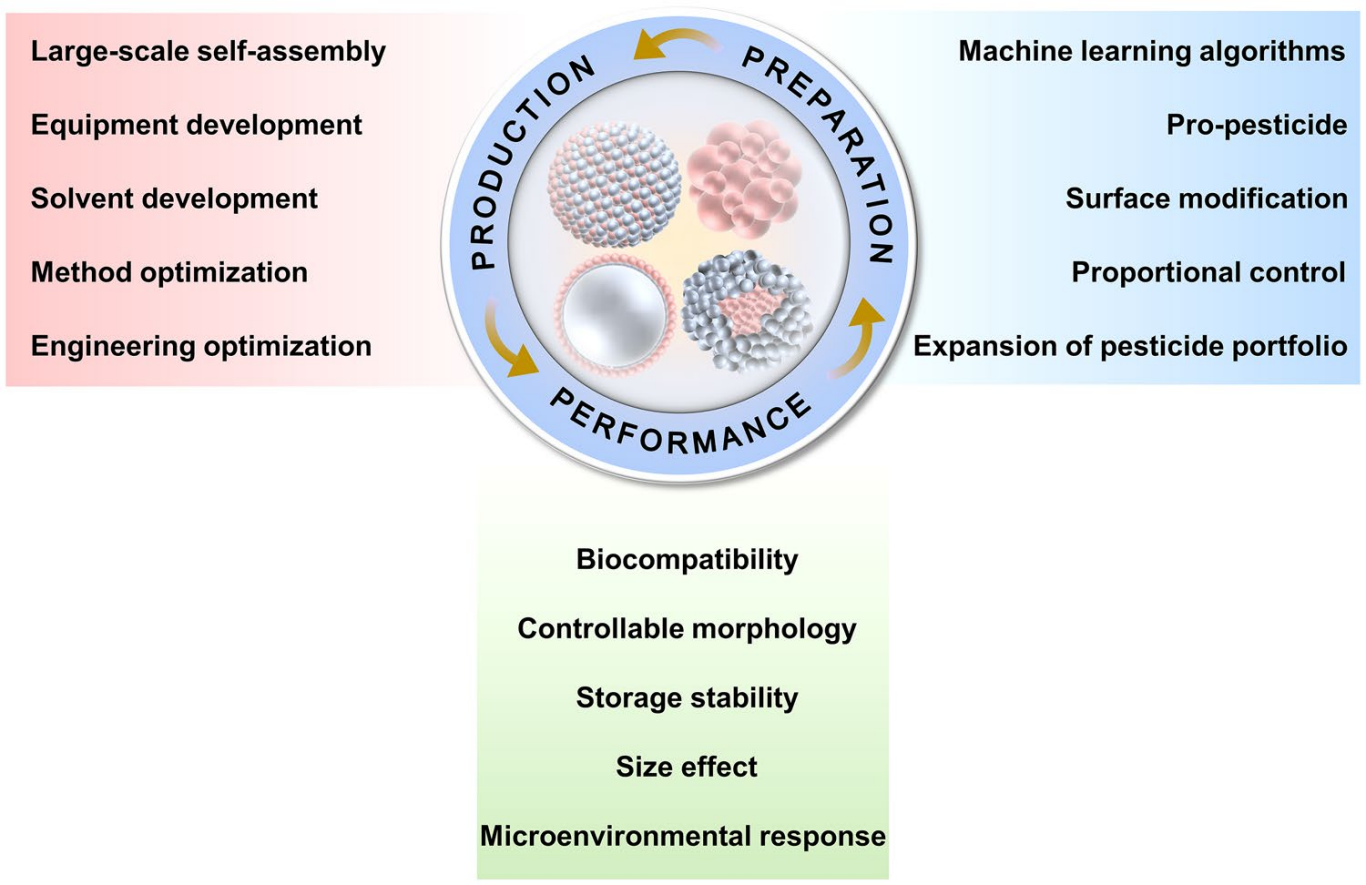
Potential directions for future research on NMC

Currently, the United Nations has incorporated the reduction the input of agrochemicals harmful to the environment and human health as one of the Sustainable Development Goals. Despite the emergence of nanopesticides as environmentally friendly alternatives to traditional pesticides, the excessive introduction of non-therapeutic or functional nanomaterials of certain research or products may pose risks to the environment and human safety. Critically, this thought has the potential to lead future research in misguided directions. Therefore, a more nuanced and comprehensive understanding are needed to discern the true benefits of extensively used nanomaterials in agrochemicals. Herein, we propose NMC that have the potential to become hot research topics. Based on the current research progress, it can be anticipated that NMC will realize widespread applications as a promising nanopesticide in the future. The significant advantages of NMC through molecular self-assembly are the preparation process that does not require excessive energy input, the extremely high loading capacity, and rich functionalization potential. However, further development is needed for NMC to attain full industrialization in the future (Fig. 6).

In terms of preparation of NMC systems, the prodrug design and molecular self-assembly stands are crucial steps, the monitoring and prediction of these reactions are necessary. Accurate quantification of the molecular structure of prodrug molecules using machine learning algorithms to predict nanoparticle formation represents a promising method to implement these steps [70]. Molecular dynamics simulations can further elucidate the formation processes of prodrug molecule and self-assembled nanoparticles, while molecular docking can investigate the molecular interactions in this process in detail [71]. These technologies are anticipated to assist chemists in elucidating the kinetic mechanism of pesticide molecule self-assembly, directly benefiting the precise synthesis of NMC systems. Controlling the proportions of molecules involved in the reaction can maximize the loading efficiency, thereby minimizing pesticide resistance in pests and bacterial caused by multiple applications. Another important significance of this study is to elucidate the synergistic effects and regulatory mechanisms between different pesticide assembly molecules. This is a field that still lacks comprehensive understanding. Establishing a more extensive portfolio of self-assembled pesticide molecules is also a research agenda that requires a significant investment of time and resources. Artificial intelligence can help reduce the cost of actual experimental operations to accelerate the development of NMC [72].

Another focus is on the performance of the NMC system. Although the developed NMC exhibit good responsive release capabilities, it remains necessary to enhance the targeting ability of NMC toward pests and fungal, bacterial, and viral diseases. With the advancement of double-stranded RNA (dsRNA) that can silence genes for plant protection, there is a growing focus on developing nano-delivery platforms to enhance the uptake efficiency of exogenous RNA by targets [73]. It is crucial to develop targeting systems capable of promoting the translocation of dsRNA across cell membranes or penetrating plant cell walls. For example, Itzhakov et al. [74] recently suggested that incorporating capryl substituents enhances the membrane penetration capability of the delivery platform. While long carbon chain molecules have been utilized in constructing NMC prodrug molecules, further discussion is needed regarding their biofilm penetration properties. The next important concept to mention is the design of prodrug molecules, which contribute to enhancing the biosafety and microenvironmental response capabilities of the NMC system [47]. Prodrugs are highly valued in medicine, especially in tumor treatment, for their ability to respond to the microenvironment in the body. And similarly, the prodrug requires further design and development based on the microenvironment when plants are exposed to various biotic or abiotic stresses [75]. It is important to note that these entirely new chemical molecules might need to undergo a comprehensive safety assessment and registration process.

The size effect has always been the motivation for the ongoing development of nanopesticides, and NMC, utilizing a bottom-up self-assembly process, facilitate the creation of an extremely small delivery platform. Based on NMC nano-platform, a systematic investigation of the interaction between it and plant cell walls will contribute to unraveling the biological mechanism of nanosystems constructed from pure pesticide molecules [76]. The loading and activity of pesticide molecules in NMC systems significantly surpasses that of NNC, which might have different effects upon contact with plant cell walls [77], and this research is obviously lacking.

Manipulating the morphology and geometry of self-assembled systems presents another promising avenue for performance exploration [78]. The favorable characteristics exhibited by single-walled carbon nanotubes in plant delivery demonstrate that nano-delivery platforms with high aspect ratios may be more readily accepted by plant cells [79]. From this perspective, the incorporation of self-assembled nanofibers or nanorods into the NMC system is anticipated to enhance its application prospects in controlling plant diseases. Alterations in the morphology and geometry of nanoparticles can also influence their overall stability. Then, the critical aspect to emphasize here is the storage stability of NMC, which is extremely important for the development of industrialized pesticide formulations. Considering the inherent instability of self-assembly systems, ongoing research primarily concentrates on regulating the solvent environment and feeding parameters during their formation. The longer-term storage stability of NMC remains insufficiently scientifically evaluated, requiring comprehensive studies across different temperature storage conditions and transportation processes.

The final consideration involves the practical production of NMC, encompassing discussions on prodrug preparation and molecular self-assembly processes. It is crucial to emphasize that the intelligent design and high-throughput synthesis of prodrug molecules are critical issues that must be addressed to achieve the industrialization of NMC. The recently suggested large-scale self-assembly approach can also serve as references for the scaled-up production of NMC [80, 81]. The effective execution of these strategies often necessitates the backing of dedicated production lines and equipment, thereby overcoming the limitations of traditional nanoprecipitation methods in terms of scalability. Microfluidic technology [62] and flash nanoprecipitation [82] methods can advance the progress of sophisticated, high-throughput, continuous-flow prodrug synthesis and molecular self-assembly. It is noteworthy that, given the necessity for cost-effective agricultural production, the optimization of NMC methods and engineering is imperative. The future emphasis will be on developing affordable production equipment of NMC system suitable for agricultural enterprises, with the possibility of downstream manipulation [83]. Additionally, future research should focus on environmentally friendly solvents that facilitate large-scale production, as the formation and overall performance of the NMC system are closely tied to the choice of solvent [84].

In summary, the development of NMC is expected to provide a new way for the future development of nanopesticides. Based on molecular structure design, NMC systems can be constructed via molecular self-assembly, which have high loading capacity, potentially high activity, good functionality, and industrialization prospects. The widespread use of NMC could help alleviate concerns regarding human health and environmental safety associated with the large-scale introduction of nanomaterials. Our perspective acknowledges that while certain NMC research has proposed viable design strategies and demonstrated promising developmental potential, addressing the remaining substantial research space and challenges in the future is crucial.

## Supplementary Information

Below is the link to the electronic supplementary material.

Supplementary file1 (DOCX 63 KB)

## References

[1] M. Zurek, A. Hebinck, O. Selomane, Climate change and the urgency to transform food systems. Science **376**, 1416–1421 (2022). 10.1126/science.abo2364 35737771 10.1126/science.abo2364

[2] F. Gould, Z.S. Brown, J. Kuzma, Wicked evolution: can we address the sociobiological dilemma of pesticide resistance? Science **360**, 728–732 (2018). 10.1126/science.aar3780 29773742 10.1126/science.aar3780

[3] R. Schulz, S. Bub, L.L. Petschick, S. Stehle, J. Wolfram, Applied pesticide toxicity shifts toward plants and invertebrates, even in GM crops. Science **372**, 81–84 (2021). 10.1126/science.abe1148 33795455 10.1126/science.abe1148

[4] J. von Braun, K. Afsana, L.O. Fresco, M. Hassan, Food systems: seven priorities to end hunger and protect the planet. Nature **597**, 28–30 (2021). 10.1038/d41586-021-02331-x 34462598 10.1038/d41586-021-02331-x

[5] T. Hofmann, G.V. Lowry, S. Ghoshal, N. Tufenkji, D. Brambilla et al., Technology readiness and overcoming barriers to sustainably implement nanotechnology-enabled plant agriculture. Nat. Food **1**, 416–425 (2020). 10.1038/s43016-020-0110-1

[6] F. Gomollón-Bel, Ten chemical innovations that will change our world: IUPAC identifies emerging technologies in chemistry with potential to make our planet more sustainable. Chem. Int. **41**, 12–17 (2019). 10.1515/ci-2019-0203

[7] D. Xiao, H. Wu, Y. Zhang, J. Kang, A. Dong et al., Advances in stimuli-responsive systems for pesticides delivery: recent efforts and future outlook. J. Control. Release **352**, 288–312 (2022). 10.1016/j.jconrel.2022.10.028 36273530 10.1016/j.jconrel.2022.10.028

[8] Q. Zhang, Y. Ying, J. Ping, Recent advances in plant nanoscience. Adv. Sci. **9**, 2103414 (2022). 10.1002/advs.202103414 10.1002/advs.202103414 PMC8805591 34761568

[9] M. Kah, N. Tufenkji, J.C. White, Nano-enabled strategies to enhance crop nutrition and protection. Nat. Nanotechnol. **14**, 532–540 (2019). 10.1038/s41565-019-0439-5 31168071 10.1038/s41565-019-0439-5

[10] A. Shelar, S.H. Nile, A.V. Singh, D. Rothenstein, J. Bill et al., Recent advances in nano-enabled seed treatment strategies for sustainable agriculture: challenges, risk assessment, and future perspectives. Nano-Micro Lett. **15**, 54 (2023). 10.1007/s40820-023-01025-5 10.1007/s40820-023-01025-5 PMC9935810 36795339

[11] R. Saberi Riseh, M. Hassanisaadi, M. Vatankhah, R.S. Varma, V.K. Thakur, Nano/micro-structural supramolecular biopolymers: innovative networks with the boundless potential in sustainable agriculture. Nano-Micro Lett. **16**, 147 (2024). 10.1007/s40820-024-01348-x 10.1007/s40820-024-01348-x PMC10923760 38457088

[12] D. Wang, N.B. Saleh, A. Byro, R. Zepp, E. Sahle-Demessie et al., Nano-enabled pesticides for sustainable agriculture and global food security. Nat. Nanotechnol. **17**, 347–360 (2022). 10.1038/s41565-022-01082-8 35332293 10.1038/s41565-022-01082-8 PMC9774002

[13] M. Kah, R.S. Kookana, A. Gogos, T.D. Bucheli, A critical evaluation of nanopesticides and nanofertilizers against their conventional analogues. Nat. Nanotechnol. **13**, 677–684 (2018). 10.1038/s41565-018-0131-1 29736032 10.1038/s41565-018-0131-1

[14] H. Flerlage, J.C. Slootweg, Modern chemistry is rubbish. Nat. Rev. Chem. **7**, 593–594 (2023). 10.1038/s41570-023-00523-9 37524899 10.1038/s41570-023-00523-9

[15] M. Kah, L.J. Johnston, R.S. Kookana, W. Bruce, A. Haase et al., Comprehensive framework for human health risk assessment of nanopesticides. Nat. Nanotechnol. **16**, 955–964 (2021). 10.1038/s41565-021-00964-7 34518657 10.1038/s41565-021-00964-7

[16] K.G. Cassman, P. Grassini, A global perspective on sustainable intensification research. Nat. Sustain. **3**, 262–268 (2020). 10.1038/s41893-020-0507-8

[17] E. Agathokleous, Z. Feng, I. Iavicoli, E.J. Calabrese, Nano-pesticides: a great challenge for biodiversity? The need for a broader perspective. Nano Today **30**, 100808 (2020). 10.1016/j.nantod.2019.100808

[18] P. Zhang, Z. Guo, Z. Zhang, H. Fu, J.C. White et al., Nanomaterial transformation in the soil-plant system: implications for food safety and application in agriculture. Small **16**, e2000705 (2020). 10.1002/smll.202000705 32462786 10.1002/smll.202000705

[19] K. Yang, Y. Li, X. Tan, R. Peng, Z. Liu, Behavior and toxicity of graphene and its functionalized derivatives in biological systems. Small **9**, 1492–1503 (2013). 10.1002/smll.201201417 22987582 10.1002/smll.201201417

[20] S.H. Lacerda, J.J. Park, C. Meuse, D. Pristinski, M.L. Becker et al., Interaction of gold nanoparticles with common human blood proteins. ACS Nano **4**, 365–379 (2010). 10.1021/nn9011187 20020753 10.1021/nn9011187

[21] S.I.L. Gomes, J.J. Scott-Fordsmand, M.J.B. Amorim, Alternative test methods for (nano)materials hazards assessment: challenges and recommendations for regulatory preparedness. Nano Today **40**, 101242 (2021). 10.1016/j.nantod.2021.101242

[22] X.-D. Sun, X.-Z. Yuan, Y. Jia, L.-J. Feng, F.-P. Zhu et al., Differentially charged nanoplastics demonstrate distinct accumulation in *Arabidopsis thaliana*. Nat. Nanotechnol. **15**, 755–760 (2020). 10.1038/s41565-020-0707-4 32572228 10.1038/s41565-020-0707-4

[23] M.F. Hochella Jr., D.W. Mogk, J. Ranville, I.C. Allen, G.W. Luther et al., Natural, incidental, and engineered nanomaterials and their impacts on the Earth system. Science **363**, eaau8299 (2019). 10.1126/science.aau8299 30923195 10.1126/science.aau8299

[24] L.M. Gilbertson, L. Pourzahedi, S. Laughton, X. Gao, J.B. Zimmerman et al., Guiding the design space for nanotechnology to advance sustainable crop production. Nat. Nanotechnol. **15**, 801–810 (2020). 10.1038/s41565-020-0706-5 32572231 10.1038/s41565-020-0706-5

[25] L. Huang, S. Zhao, F. Fang, T. Xu, M. Lan et al., Advances and perspectives in carrier-free nanodrugs for cancer chemo-monotherapy and combination therapy. Biomaterials **268**, 120557 (2021). 10.1016/j.biomaterials.2020.120557 33260095 10.1016/j.biomaterials.2020.120557

[26] H. Mei, S. Cai, D. Huang, H. Gao, J. Cao et al., Carrier-free nanodrugs with efficient drug delivery and release for cancer therapy: from intrinsic physicochemical properties to external modification. Bioact. Mater. **8**, 220–240 (2021). 10.1016/j.bioactmat.2021.06.035 34541398 10.1016/j.bioactmat.2021.06.035 PMC8424425

[27] S. Karaosmanoglu, M. Zhou, B. Shi, X. Zhang, G.R. Williams et al., Carrier-free nanodrugs for safe and effective cancer treatment. J. Control. Release **329**, 805–832 (2021). 10.1016/j.jconrel.2020.10.014 33045313 10.1016/j.jconrel.2020.10.014

[28] L.-H. Liu, X.-Z. Zhang, Carrier-free nanomedicines for cancer treatment. Prog. Mater. Sci. **125**, 100919 (2022). 10.1016/j.pmatsci.2021.100919

[29] D. Liu, R. Aleisa, Z. Cai, Y. Li, Y. Yin, Self-assembly of superstructures at all scales. Matter **4**, 927–941 (2021). 10.1016/j.matt.2020.12.020

[30] E.L. Etter, K.-C. Mei, J. Nguyen, Delivering more for less: nanosized, minimal-carrier and pharmacoactive drug delivery systems. Adv. Drug Deliv. Rev. **179**, 113994 (2021). 10.1016/j.addr.2021.113994 34619287 10.1016/j.addr.2021.113994 PMC9633093

[31] Y. Su, X. Zhou, H. Meng, T. Xia, H. Liu et al., Cost-benefit analysis of nanofertilizers and nanopesticides emphasizes the need to improve the efficiency of nanoformulations for widescale adoption. Nat. Food **3**, 1020–1030 (2022). 10.1038/s43016-022-00647-z 37118298 10.1038/s43016-022-00647-z

[32] I. Mubeen, M. Fawzi Bani Mfarrej, Z. Razaq, S. Iqbal, S.A.H. Naqvi et al., Nanopesticides in comparison with agrochemicals: outlook and future prospects for sustainable agriculture. Plant Physiol. Biochem. **198**, 107670 (2023). 10.1016/j.plaphy.2023.107670 37018866 10.1016/j.plaphy.2023.107670

[33] N. Kandhol, V.P. Singh, L. Herrera-Estrella, L.P. Tran, D.K. Tripathi, Nanocarrier spray: a nontransgenic approach for crop engineering. Trends Plant Sci. **28**, 259–261 (2023). 10.1016/j.tplants.2022.12.015 36585337 10.1016/j.tplants.2022.12.015

[34] M. Li, Z. Ma, M. Peng, L. Li, M. Yin et al., A gene and drug co-delivery application helps to solve the short life disadvantage of RNA drug. Nano Today **43**, 101452 (2022). 10.1016/j.nantod.2022.101452

[35] D.-X. Zhang, J. Du, R. Wang, J. Luo, T.-F. Jing et al., Core/shell dual-responsive nanocarriers via iron-mineralized electrostatic self-assembly for precise pesticide delivery. Adv. Funct. Mater. **31**, 2102027 (2021). 10.1002/adfm.202102027

[36] J. Luo, Y. Gao, Y. Liu, X. Huang, D.-X. Zhang et al., Self-assembled degradable nanogels provide foliar affinity and pinning for pesticide delivery by flexibility and adhesiveness adjustment. ACS Nano **15**, 14598–14609 (2021). 10.1021/acsnano.1c04317 34427447 10.1021/acsnano.1c04317

[37] G.M. Whitesides, M. Boncheva, Beyond molecules: self-assembly of mesoscopic and macroscopic components. Proc. Natl. Acad. Sci. U.S.A. **99**, 4769–4774 (2002). 10.1073/pnas.082065899 11959929 10.1073/pnas.082065899 PMC122665

[38] J. Kim, S. Lee, Y. Kim, M. Choi, I. Lee et al., *In situ* self-assembly for cancer therapy and imaging. Nat. Rev. Mater. **8**, 710–725 (2023). 10.1038/s41578-023-00589-3

[39] K. Wang, Y. Wang, Y. Wu, J. Jiang, Y. Zhang et al., A novel dual stimuli-responsive and double-loaded insecticidal nanoformulation for efficient control of insect pest. Chem. Eng. J. **474**, 146012 (2023). 10.1016/j.cej.2023.146012

[40] X. Li, Z. Zhou, Y. Huang, G. Tang, Y. Liu et al., A high adhesion co-assembly based on myclobutanil and tannic acid for sustainable plant disease management. Pest Manag. Sci. **79**, 3796–3807 (2023). 10.1002/ps.7564 37209275 10.1002/ps.7564

[41] G. Gohari, M. Jiang, G.A. Manganaris, J. Zhou, V. Fotopoulos, Next generation chemical priming: with a little help from our nanocarrier friends. Trends Plant Sci. **29**, 150–166 (2024). 10.1016/j.tplants.2023.11.024 38233253 10.1016/j.tplants.2023.11.024

[42] Y. Zhang, G.G. Goss, Nanotechnology in agriculture: comparison of the toxicity between conventional and nano-based agrochemicals on non-target aquatic species. J. Hazard. Mater. **439**, 129559 (2022). 10.1016/j.jhazmat.2022.129559 35863222 10.1016/j.jhazmat.2022.129559

[43] R. Pires-Oliveira, M.S. Kfouri, B. Mendonça, P. Cardoso-Gustavson, Nanopesticides: From the Bench to the Market, in *Nanopesticides*. ed. by L.F. Fraceto, V.L.S.S. de Castro, R. Grillo, D. Ávila, H.C. Oliveira et al. (Springer, Cham, 2020), pp.317–348

[44] C. An, B. Huang, J. Jiang, X. Wang, N. Li et al., Design and synthesis of a water-based nanodelivery pesticide system for improved efficacy and safety. ACS Nano **18**, 662–679 (2024). 10.1021/acsnano.3c08854 38134332 10.1021/acsnano.3c08854

[45] Q.-T. Ji, D.-K. Hu, X.-F. Mu, X.-X. Tian, L. Zhou et al., Cucurbit[7]uril-mediated supramolecular bactericidal nanoparticles: their assembly process, controlled release, and safe treatment of intractable plant bacterial diseases. Nano Lett. **22**, 4839–4847 (2022). 10.1021/acs.nanolett.2c01203 35667033 10.1021/acs.nanolett.2c01203

[46] X. Chi, G. Yu, L. Shao, J. Chen, F. Huang, A dual-thermoresponsive gemini-type supra-amphiphilic macromolecular[3]pseudorotaxane based on pillar[10]arene/paraquat cooperative complexation. J. Am. Chem. Soc. **138**, 3168–3174 (2016). 10.1021/jacs.5b13173 26862921 10.1021/jacs.5b13173

[47] X. Zhang, G. Tang, Z. Zhou, H. Wang, X. Li et al., Fabrication of enzyme-responsive prodrug self-assembly based on fluazinam for reducing toxicity to aquatic organisms. J. Agric. Food Chem. **71**, 12678–12687 (2023). 10.1021/acs.jafc.3c03762 37595273 10.1021/acs.jafc.3c03762

[48] Y. Tian, G. Tang, Y. Gao, X. Chen, Z. Zhou et al., Carrier-free small molecular self-assembly based on berberine and curcumin incorporated in submicron particles for improving antimicrobial activity. ACS Appl. Mater. Interfaces **14**, 10055–10067 (2022). 10.1021/acsami.1c22900 35175042 10.1021/acsami.1c22900

[49] B. Testa, Prodrug research: futile or fertile? Biochem. Pharmacol. **68**, 2097–2106 (2004). 10.1016/j.bcp.2004.07.005 15498500 10.1016/j.bcp.2004.07.005

[50] K. Wei, Z. Li, Z. Zheng, Y. Gao, Q. Huang et al., Natural glycyrrhizic acid-tailored nanoparticles toward the enhancement of pesticide bioavailability. Adv. Funct. Mater. (2024). 10.1002/adfm.202315493

[51] Y. Tian, Y. Huang, X. Zhang, G. Tang, Y. Gao et al., Self-assembled nanoparticles of a prodrug conjugate based on pyrimethanil for efficient plant disease management. J. Agric. Food Chem. **70**, 11901–11910 (2022). 10.1021/acs.jafc.2c04489 36111893 10.1021/acs.jafc.2c04489

[52] Y. Tian, X. Zhang, Y. Huang, G. Tang, Y. Gao et al., Amphiphilic prodrug nano-micelles of fipronil coupled with natural carboxylic acids for improving physicochemical properties and reducing the toxicities to aquatic organisms. Chem. Eng. J. **439**, 135717 (2022). 10.1016/j.cej.2022.135717

[53] K. Zhao, G. Xu, L. Wang, T. Wu, X. Zhang et al., Using a dynamic hydrophilization strategy to achieve nanodispersion, full wetting, and precise delivery of hydrophobic pesticide. ACS Appl. Mater. Interfaces **15**, 37093–37106 (2023). 10.1021/acsami.3c07530 37488063 10.1021/acsami.3c07530

[54] Q. Liu, B. Graham, A. Hawley, Y.-D. Dong, B.J. Boyd, Novel agrochemical conjugates with self-assembling behaviour. J. Colloid Interface Sci. **512**, 369–378 (2018). 10.1016/j.jcis.2017.10.070 29096098 10.1016/j.jcis.2017.10.070

[55] P.P. Nadiminti, Y.D. Dong, C. Sayer, P. Hay, J.E. Rookes et al., Nanostructured liquid crystalline particles as an alternative delivery vehicle for plant agrochemicals. ACS Appl. Mater. Interfaces **5**, 1818–1826 (2013). 10.1021/am303208t 23421455 10.1021/am303208t

[56] J.-G. Cui, D.-M. Mo, Y. Jiang, C.-F. Gan, W.-G. Li et al., Fabrication, characterization, and insecticidal activity evaluation of emamectin benzoate–sodium lignosulfonate nanoformulation with pH-responsivity. Ind. Eng. Chem. Res. **58**, 19741–19751 (2019). 10.1021/acs.iecr.9b03171

[57] Y. Xiao, C. Wu, P. Cui, X. Luo, L. Zhou et al., Enhancing adsorption capacity and herbicidal efficacy of 2, 4-D through supramolecular self-assembly: insights from cocrystal engineering to solution chemistry. Chem. Eng. J. **469**, 143757 (2023). 10.1016/j.cej.2023.143757

[58] Y. Tian, G. Tang, Y. Li, Z. Zhou, X. Chen et al., A simple preparation process for an efficient nano-formulation: small molecule self-assembly based on spinosad and sulfamic acid. Green Chem. **23**, 4882–4891 (2021). 10.1039/D1GC00971K

[59] J. Murray, K. Kim, T. Ogoshi, W. Yao, B.C. Gibb, The aqueous supramolecular chemistry of cucurbit[*n*]urils, pillar[*n*]arenes and deep-cavity cavitands. Chem. Soc. Rev. **46**, 2479–2496 (2017). 10.1039/c7cs00095b 28338130 10.1039/c7cs00095b PMC5462124

[60] T.L. Price Jr., H.W. Gibson, Supramolecular pseudorotaxane polymers from biscryptands and bisparaquats. J. Am. Chem. Soc. **140**, 4455–4465 (2018). 10.1021/jacs.8b01480 29510043 10.1021/jacs.8b01480

[61] Q. Song, L. Mei, X. Zhang, P. Xu, M.K. Dhinakaran et al., Spreading of benquitrione droplets on superhydrophobic leaves through pillar[5]arene-based host-guest chemistry. Chem. Commun. **56**, 7593–7596 (2020). 10.1039/d0cc02187c 10.1039/d0cc02187c 32514516

[62] Y. Zheng, Z. Yu, R.M. Parker, Y. Wu, C. Abell et al., Interfacial assembly of dendritic microcapsules with host-guest chemistry. Nat. Commun. **5**, 5772 (2014). 10.1038/ncomms6772 25511608 10.1038/ncomms6772

[63] C. Gao, Q. Huang, Q. Lan, Y. Feng, F. Tang et al., A user-friendly herbicide derived from photo-responsive supramolecular vesicles. Nat. Commun. **9**, 2967 (2018). 10.1038/s41467-018-05437-5 30054483 10.1038/s41467-018-05437-5 PMC6063903

[64] J. Yang, H.-J. Ye, H.-M. Xiang, X. Zhou, P.-Y. Wang et al., Photo-stimuli smart supramolecular self-assembly of azobenzene/*β*-cyclodextrin inclusion complex for controlling plant bacterial diseases. Adv. Funct. Mater. **33**, 2303206 (2023). 10.1002/adfm.202303206

[65] Q.-T. Ji, X.-F. Mu, D.-K. Hu, L.-J. Fan, S.-Z. Xiang et al., Fabrication of host-guest complexes between adamantane-functionalized 1, 3, 4-oxadiazoles and *β*-cyclodextrin with improved control efficiency against intractable plant bacterial diseases. ACS Appl. Mater. Interfaces **14**, 2564–2577 (2022). 10.1021/acsami.1c19758 34981928 10.1021/acsami.1c19758

[66] G. Tang, Z. Zhou, X. Zhang, Y. Liu, G. Yan et al., Fabrication of supramolecular self-assembly of the schiff base complex for improving bioavailability of aldehyde-containing plant essential oil. Chem. Eng. J. **471**(1), 144471 (2023). 10.1016/j.cej.2023.144471

[67] G. Tang, Y. Tian, J. Niu, J. Tang, J. Yang et al., Development of carrier-free self-assembled nanoparticles based on fenhexamid and polyhexamethylene biguanide for sustainable plant disease management. Green Chem. **23**, 2531–2540 (2021). 10.1039/D1GC00006C

[68] H. Wang, G. Tang, Z. Zhou, X. Chen, Y. Liu et al., Stable fluorescent nanoparticles based on Co-assembly of acifluorfen and Poly (salicylic acid) for enhancing herbicidal activity and reducing environmental risks. ACS Appl. Mater. Interfaces **15**, 4303–4314 (2023). 10.1021/acsami.2c18642 36631294 10.1021/acsami.2c18642

[69] G. Tang, Y. Tian, Y. Gao, Z. Zhou, X. Chen et al., Supramolecular self-assembly of herbicides with reduced risks to the environment. ACS Nano **16**, 4892–4904 (2022). 10.1021/acsnano.2c00539 35191690 10.1021/acsnano.2c00539

[70] Y. Shamay, J. Shah, M. Işık, A. Mizrachi, J. Leibold et al., Quantitative self-assembly prediction yields targeted nanomedicines. Nat. Mater. **17**, 361–368 (2018). 10.1038/s41563-017-0007-z 29403054 10.1038/s41563-017-0007-z PMC5930166

[71] B. Sun, C. Luo, X. Zhang, M. Guo, M. Sun et al., Probing the impact of sulfur/selenium/carbon linkages on prodrug nanoassemblies for cancer therapy. Nat. Commun. **10**, 3211 (2019). 10.1038/s41467-019-11193-x 31324811 10.1038/s41467-019-11193-x PMC6642185

[72] R.L. Greenaway, K.E. Jelfs, A.C. Spivey, S.N. Yaliraki, From alchemist to AI chemist. Nat. Rev. Chem. **7**, 527–528 (2023). 10.1038/s41570-023-00522-w 37488249 10.1038/s41570-023-00522-w

[73] Y. Wang, M. Li, J. Ying, J. Shen, D. Dou et al., High-efficiency green management of potato late blight by a self-assembled multicomponent nano-bioprotectant. Nat. Commun. **14**, 5622 (2023). 10.1038/s41467-023-41447-8 37699893 10.1038/s41467-023-41447-8 PMC10497615

[74] R. Itzhakov, H. Hak, S. Sadhasivam, E. Belausov, E. Fallik et al., Nanogel particles based on modified nucleosides and oligosaccharides as advanced delivery system. ACS Nano **17**, 23020–23031 (2023). 10.1021/acsnano.3c08627 37934119 10.1021/acsnano.3c08627

[75] W. Han, X.-Q. Xu, X. Lian, Y. Chu, Y. Wang, A degradable quaternary ammonium-based pesticide safe for humans. CCS Chem. (2023). 10.31635/ccschem.023.202303338

[76] L. Zhu, W. Xu, X. Yao, L. Chen, G. Li et al., Cell wall pectin content refers to favored delivery of negatively charged carbon dots in leaf cells. ACS Nano **17**, 23442–23454 (2023). 10.1021/acsnano.3c05182 37991776 10.1021/acsnano.3c05182

[77] P. Hu, J. An, M.M. Faulkner, H. Wu, Z. Li et al., Nanoparticle charge and size control foliar delivery efficiency to plant cells and organelles. ACS Nano **14**, 7970–7986 (2020). 10.1021/acsnano.9b09178 32628442 10.1021/acsnano.9b09178

[78] J. Lv, X. Gao, B. Han, Y. Zhu, K. Hou et al., Self-assembled inorganic chiral superstructures. Nat. Rev. Chem. **6**, 125–145 (2022). 10.1038/s41570-021-00350-w 37117298 10.1038/s41570-021-00350-w

[79] S.Y. Kwak, T.T.S. Lew, C.J. Sweeney, V.B. Koman, M.H. Wong et al., Chloroplast-selective gene delivery and expression in planta using chitosan-complexed single-walled carbon nanotube carriers. Nat. Nanotechnol. **14**, 447–455 (2019). 10.1038/s41565-019-0375-4 30804482 10.1038/s41565-019-0375-4

[80] D. Lin, Y. Li, Large-scale 2D-confined self-assembly of colloidal nanoparticles via dynamic ice crystal templates. ACS Cent. Sci. **8**, 510–512 (2022). 10.1021/acscentsci.2c00531 35647278 10.1021/acscentsci.2c00531 PMC9136964

[81] H. Huang, J. Li, M. Yuan, H. Yang, Y. Zhao et al., Large-scale self-assembly of MOFs colloidosomes for bubble-propelled micromotors and stirring-free environmental remediation. Angew. Chem. Int. Ed. Engl. **61**, e202211163 (2022). 10.1002/anie.202211163 36121046 10.1002/anie.202211163

[82] J. Tang, X. Tong, Y. Chen, Y. Wu, Z. Zheng et al., Deposition and water repelling of temperature-responsive nanopesticides on leaves. Nat. Commun. **14**, 6401 (2023). 10.1038/s41467-023-41878-3 37828020 10.1038/s41467-023-41878-3 PMC10570302

[83] C.K. Wong, R.Y. Lai, M.H. Stenzel, Dynamic metastable polymersomes enable continuous flow manufacturing. Nat. Commun. **14**, 6237 (2023). 10.1038/s41467-023-41883-6 37802997 10.1038/s41467-023-41883-6 PMC10558441

[84] G. Moreno-Alcántar, A. Aliprandi, R. Rouquette, L. Pesce, K. Wurst et al., Solvent-driven supramolecular wrapping of self-assembled structures. Angew. Chem. Int. Ed. **60**, 5407–5413 (2021). 10.1002/anie.202013474 10.1002/anie.202013474 PMC7986396 33247479

