# Correction: Making the Complicated Simple: A Minimizing Carrier Strategy on Innovative Nanopesticides

**DOI:** 10.1007/s40820-024-01460-y

**Published:** 2024-07-01

**Authors:** Wenjie Shangguan, Qiliang Huang, Huiping Chen, Yingying Zheng, Pengyue Zhao, Chong Cao, Manli Yu, Yongsong Cao, Lidong Cao

**Affiliations:** 1 State Key Laboratory for Biology of Plant Diseases and Insect Pests, Institute of Plant Protection, Chinese Academy of Agricultural Sciences, Beijing, 100193 People’s Republic of China; 2 College of Plant Protection, China Agricultural University, Beijing, 100193 People’s Republic of China; 3 State Key Laboratory of Element‑Organic Chemistry, Department of Chemical Biology, College of Chemistry, Nankai University, Tianjin, 300071 People’s Republic of China

**Correction to: Nano-Micro Lett. (2024) 16:193** 10.1007/s40820-024-01413-5

The Nano-Micro Letters (2024) 16:193, article by Shangguan et al., entitled “Making the Complicated Simple: A Minimizing Carrier Strategy on Innovative Nanopesticides” (Nano-Micro Lett. 10.1007/s40820-024-01413-5), was published online on 14 May, 2024, with errors.

The structural formulas and captions of the three acyl chlorides in Fig. 3A were wrong. They should be as shown below.

**Fig. 3:**
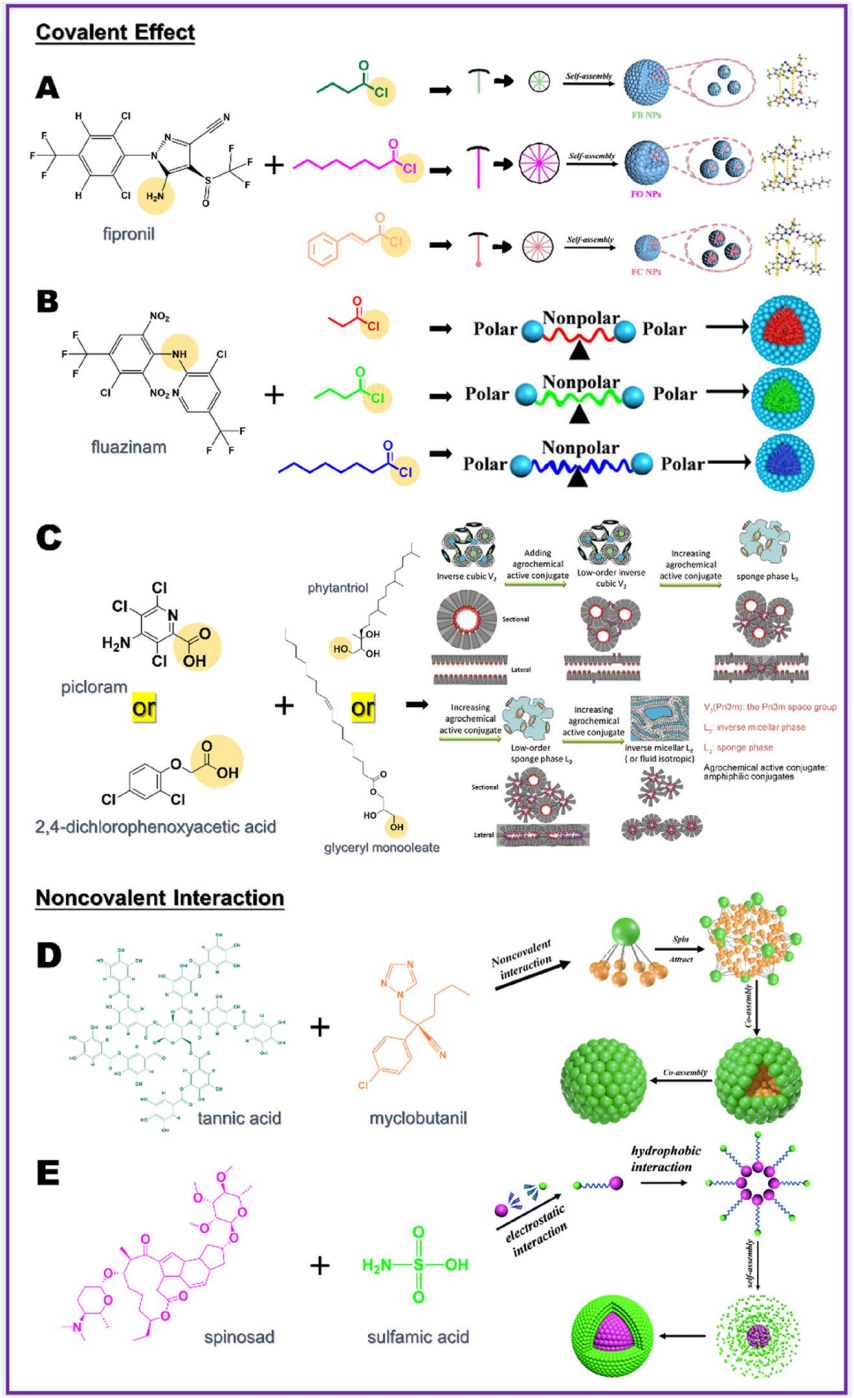
NMC preparation strategy based on the interaction between small molecules. **A** Schematic illustration of fipronil and three acyl chlorides forming NMC based on amide bond [52], copyright 2022, Elsevier. **B** Schematic illustration of fluazinam and three acyl chlorides forming NMC based on amide bond [47], copyright 2023, American Chemical Society. **C** Schematic illustration of 2,4-dichlorophenoxyacetic acid or picloraml and phytantriol or glyceryl monooleate forming NMC based on ester bond [54], copyright 2018, Elsevier. **D** Schematic illustration of myclobutanil and tannic acid forming NMC based on noncovalent interaction [40], copyright 2023, Wiley Online. **E** Schematic illustration of spinosad and sulfamic acid forming NMC based on noncovalent interaction [58], copyright 2021, Royal Society of Chemistry

We have carefully reviewed the figures and text in the original article and found inconsistencies. We apologize for not identifying these issues earlier.

To ensure the accuracy of scientific information, we have revised the structural formulas and updated the figure captions accordingly.

The corrected figure and caption are as follows.

The revised Fig. 3.

