# Supplementary material for: Making the Complicated Simple: A Minimizing Carrier Strategy on Innovative Nanopesticides
Source: Nanomicro Lett. 2024 May 14;16:193. doi: 10.1007/s40820-024-01413-5 (PMC11093950; doi:10.1007/s40820-024-01413-5)
Supplement: Supplementary file 1 — Supplementary file1 (DOCX 63 KB) [file 40820_2024_1413_MOESM1_ESM.docx]

Supporting Information for

**Making the Complicated Simple: A Minimizing Carrier Strategy on Innovative Nanopesticides**

Wenjie Shangguan^1^, Qiliang Huang^1,^*, Huiping Chen^1^, Yingying Zheng^1,3^, Pengyue Zhao^1^, Chong Cao^1^, Manli Yu^1^, Yongsong Cao^2,^* and Lidong Cao^1,^*

^1^ State Key Laboratory for Biology of Plant Diseases and Insect Pests,Institute of Plant Protection, Chinese Academy of Agricultural Sciences, Beijing 100193, P. R. China

^2^ College of Plant Protection, China Agricultural University,Beijing 100193, P. R. China

^3^ State Key Laboratory of Element-Organic Chemistry,Department of Chemical Biology, College of Chemistry, Nankai University, Tianjin 300071, P. R. China

*Corresponding authors. E-mail: [qlhuang@ippcaas.cn](mailto:qlhuang@ippcaas.cn) (Qiliang Huang); [caoys@cau.edu.cn](mailto:caoys@cau.edu.cn) (Yongsong Cao); [caolidong@caas.cn](mailto:caolidong@caas.cn) (Lidong Cao)

**Supplementary Tables**

**Abbreviations**: EC_50_: concentrations that have an effect at 50% of test organisms; IC_50_: concentrations that inhibit 50% of test organisms; LC_50_: Concentrations that kill 50% of test organisms; LE: loading efficiency; PDI: polydispersity index; DT_50_: Half-life of the degradation; DST: Dynamic surface tension; SAR: Self-assembly rate.

**Table S1** Physicochemical Properties in different NMC and NNC references

| Symbol | Type | Nanocarrier of NNC/  Molecule of NMC | Active Ingredient | Physicochemical Properties | Refs. |
| --- | --- | --- | --- | --- | --- |
| 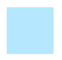 | NMC | Propionyl chloride (FZNP1), butyryl chloride (FZNP2), and octanoyl chloride (FZNP3) | Fluazinam (Flu) | PDI, Z-Average, Zeta potentials of FZNP1, FZNP2 and FZNP3 were 0.077, 366 nm and -28.9 mV; 0.072, 324 nm and -33.7 mV; 0.043, 232 nm and -34.5 mV  DT_50_, DST and yields of FZNP1, FZNP2 and FZNP3 were 182.62 min, 57.42 mN/m and 78.21%; 206.10 min, 55.516 mN/m and 74.83%; 572.31 min, 53.690 mN/m and 69.78% | [S1] |
| 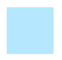 | NMC | Branched polyethylenimine (BPEI) with an average molecular weight of 10,000 | Cinnamaldehyde (Cin) | PDI and Z-Average of Cin/BPEI were 0.217 and 218 nm  DST of Cin/BPEI was 70.82 mN/m  SAR of Cin/BPEI > 90% | [S2] |
| 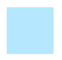 | NMC | Sulfamic acid (SA) | Spinosad (SSD) | PDI, Z-Average, Zeta potentials of SSD-SA were 0.084, 7.740 nm and +47.8 mV  DST of SSD-SA was 34.60 mN/m  SAR of SSD-SA (SA:SSD = 1 g : 3 g; pH = 2; 25°C; Ionic concentration = 100 mmol/L) was 87.94% | [S3] |
| 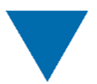 | NNC | Polydopamine surface modified MXene-Ti_3_C_2_T_x_ (PDA@Ti_3_C_2_T_x_) | Emamectin benzoate (EB) | Photothermal conversion effect of EB@PDA@Ti_3_C_2_T_x_ was 34.5%  LE of EB@PDA@Ti_3_C_2_T_x_ was 45.37% | [S4] |
| 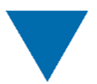 | NNC | Lanthanum-modified chitosan oligosaccharide (Cos-La) | Avermectin (AVM) | Particle sizes and PDI of AVM-loaded Cos-La were 333.1 nm and 0.425  LE of AVM-loaded Cos-La was 46.3% | [S5] |
| 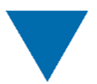 | NNC | Chitin nanocrystals and Cu (ChNC@Cu) | Cu^2+^ | ChNC@Cu was 271.3 ± 10.2 nm long and 37.2 ± 4.7 nm wide  Zeta potential of ChNC@Cu was -16.2 mV  LE of ChNC@Cu was 2.63% | [S6] |
| 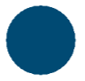 | NNC | Mesoporous silica nanoparticles (MSNs) | Spirotetramat (Stm) | Particle sizes of Stm@MSNs were 112.5 nm (Stm@MSNs-100), 200.1 nm (Stm@MSNs-200), and 439.4 nm (Stm@MSNs-400)  LE of Stm@MSNs-100, Stm@MSNs-200, and Stm@MSNs-400 were 38%, 21%, and 53% | [S7] |
| 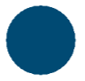 | NNC | Silicon-based iron-doped nanoparticle (Fe-MSN) and tannic acid (TA) | Prochloraz (Pro) | Particle sizes, Zeta potentials and PDI of Pro@Fe-MSNs/TA were 471.1 nm, −42.8 and 0.37  LE of Pro@Fe-MSNs/TA was 31% | [S8] |
| 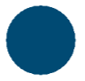 | NNC | MSNs | Pyraclostrobin (Py) | Particle size of Py@MSNs was 410 nm  LE of Py@MSNs was 28.5% | [S9] |
| 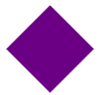 | NNC | Zein-functionalized mesoporous silica (MSN-SS-Zein) | Avermectin (AVM) | Particle size and Zeta potentials of MSN-SS-Zein were 150 nm and -16.43 to 31.7 mV  LE of MSN-SS-Zein was 22.4% | [S10] |
| 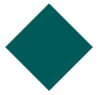 | NNC | Castor oil-based polyurethanes (CO-PU) | Avermectin (AVM) | Particle sizes, PDI and Zeta potentials of AVM/CO-PU were 45.5 to 48.8 nm, 0.215 to 0.311 and -34.3 to -29.7 mV  LE of AVM/CO-PU was 42.3% | [S11] |
| 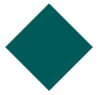 | NNC | Polylactide (PLA), bovine serum albumin (BSA) and poly(vinyl alcohol) (PVA) | Chlorantraniliprole (CAP) | Particle sizes of Microcapsules were 4.2 to 0.65 μm  LE of Microcapsules was 31% | [S12] |
| 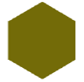 | NNC | Ethidium bromide-based covalent organic frameworks (EB-COFs) | Quinclorac (QNC) | LE of QNC into EB-COFs was 41% | [S13] |
| 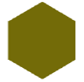 | NNC | Zeolitic Imidazole Framework-90 (ZIF-90) | Kasugamycin (KSM) | Particle sizes, DT_50_ and PDI of ZIF-90-KSM were 100 to 200 nm, 37.46 h and -9.6 mV  LE of ZIF-90-KSM was 6.7% | [S14] |
| 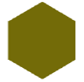 | NNC | Zeolitic imidazolate framework-8 composite (ZIF-8) | Dazomet (DZ) | Particle size of DZ@ZIF-8 was 71.5 nm  LE of DZ@ZIF-8 was 4.425% | [S15] |
| 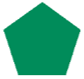 | NNC | Carboxymethyl starch with metal-organic frameworks (MIL-101(Fe)-CMS) | Chlorantraniliprole (CAP) | Zeta potential of CAP@MIL-101(Fe)-CMS was -20.60 mV  LE of CAP@MIL-101(Fe)-CMS was 18.3% | [S16] |

**Table S2** Target organism toxicity in different NMC and NNC references

| Type | Nanocarrier of NNC/  Molecule of NMC | Active Ingredient | Target organism | Toxicity | Refs. |
| --- | --- | --- | --- | --- | --- |
| NMC | Butyric acid | Pyrimethanil | *Sclerotinia sclerotiorum* | EC_50_ was 0.77 mg/L | [S17] |
| NMC | Sulfamic acid | Spinosad | *Plutella xylostella* | LC_50_ was 1.036 mg/L at 72 h | [S3] |
| NMC | Butyryl chloride/ Octanoyl chloride/ Cinnamoyl chloride | Fipronil | *Plutella xylostella* | LC_50_ were 0.172, 0.443, and 0.122 mg/L at 120 h respectively | [S18] |
| NMC | Glycyrrhizic acid | Spinosad | *Plutella xylostella* | LC_50_ was 0.38 mg/L at 48 h | [S19] |
| NMC | β-cyclodextrin–adamantane | 1,3,4-oxadiazole | *Xanthomonas oryzae pv. Oryzae*/ *Xanthomonas axonopodis pv. Citri*/ *Pseudomonas syringae pv. actinidiae* | EC_50_ were 1.04, 1.50, and 5.21 μg/mL respectively | [S20] |
| NMC | β-cyclodextrin | Azobenzene derivative | *Xanthomonas oryzae pv. Oryzae* | EC_50_ was 1.35 μg/mL at UV | [S21] |
| NMC | / | Fenhexamid and polyhexamethylene biguanide | *Botrytis cinerea*/*Sclerotinia sclerotiorum* | EC_50_ were 3.26 and 0.18 mg/L respectively | [S22] |
| NNC | Silicon-based iron-doped nanoparticle and tannic acid | Prochloraz | *Rhizoctonia solani* | IC_50_ was 0.24 μg/mL | [S8] |
| NNC | Polylactide, bovine serum albumin and poly(vinyl alcohol) | Chlorantraniliprole | *Plutella xylostella* | LC_50_ was 3.3 μg/mL | [S12] |
| NNC | Zeolitic Imidazole Framework-90 | Kasugamycin | *Magnaporthe oryzae* | EC_50_ was 2.33 mg/L | [S14] |
| NNC | Zeolitic imidazolate framework-8 composite | Dazomet | *Botrytis cinerea* | EC_50_ was 7.9 mg/L | [S15] |
| NNC | Zein-functionalized mesoporous silica | Avermectin | *Plutella xylostella* | LC_50_ was 8.21 mg/L | [S10] |
| NNC | Carboxymethyl starch with metal-organic frameworks | Chlorantraniliprole | *Spodoptera frugiperda larvae* | LC_50_ was 0.99 mg/L | [S16] |
| NNC | Metal-phenolic networks | Pyraclostrobin and thiophanate-methyl | *Botrytis cinerea* | EC_50_ was 0.145 mg/L | [S23] |
| NNC | Cellulose nanocrystals, poly(lactic acid) and methoxylated sucrose soyate polyols | Azoxystrobin | *Sclerotinia sclerotiorum/Sclerotinia sclerotiorum isolate by Muñoz.* | EC_50_ were 1.10 and 0.29 μg/mL respectively | [S24] |
| NNC | Carboxylated β-cyclodextrin and hollow mesoporous silica | Indoxacarb | *Spodoptera frugiperda* | EC_50_ were 3.61, 4.02, 5.09 and 6.98 mg/L at 0, 3, 7 and 14 day respectively | [S25] |
| NNC | Metal–organic framework MIL-101 and tannic acid | Tebuconazole | *Rhizoctonia solani/Fusarium graminearum* | EC_50_ were 0.1566 and 0.2037 mg/L respectively | [S26] |
| NNC | Zeolitic imidazolate framework-8 composite | β-cypermethrin | *Coptotermes formosanus* Shiraki | LC_50_ were 3.164, 2.569, 3.068 and 3.747 mg/L at 1, 3, 7 and 14 day respectively | [S27] |
| NNC | Poly(N-isopropylacrylamide) | Lambda-cyhalothrin | *Plutella xylostella* | LC_50_ were 5.67 and 3.02 mg/L at 25 and 29 °C respectively | [S28] |
| NNC | Zeolitic imidazolate framework-8 composite | Prochloraz | *Sclerotinia sclerotiorum* | EC_50_ were 0.122 and 0.278 mg/L at light and dark respectively | [S29] |
| NNC | Graphene oxide/ultrasonic graphene oxide | Carbendazim | *Magnaporthe oryzae* | EC_50_ were 0.28 and 0.41 mg/L respectively | [S30] |

**Table S3** Target organism toxicity in different NMC and NNC references

| Type | Number of values | Mean | Minimum | Maximum | 25% Perc. | 75% Perc. | 10% Perc. | 90% Perc. | Median | standard deviation | Refs. |
| --- | --- | --- | --- | --- | --- | --- | --- | --- | --- | --- | --- |
| NNC | 25 | 1.90686 | 0.1218 | 8.19672 | 0.26688 | 3.44828 | 0.14327 | 6.3857 | 0.38926 | 2.41517 | [S3, S17-S22] |
| NMC | 12 | 2.46556 | 0.19194 | 8.19672 | 0.7037 | 4.09357 | 0.30675 | 5.81395 | 1.13198 | 2.62146 | [S8, S10, S12, S14-S16, S23-S30] |

Note: The data were extracted from dose-response relationships, focusing on parameters EC_50_, IC_50_, and LC_50_, which directly correlate with lethal and inhibitory effects. Since these parameters were negatively related to toxicity, the inverse ratio was chosen as the calculated data. This analysis method refers to part of the analysis article published by Kah et al. [31]. The toxicithy data of NNC are lower than the findings of Kah et al. (2018) and Wang et al. (2022) [32]. Because their research involved the addition of metal-based nanomaterials (such as Ag, Ti, Cu, etc.) to nanopesticide systems, these nanomaterials have a potent effect in inducing cytotoxicity and cell death [33]. Additionally, nanoemulsions, formulations comprising significant quantities of organic solvents, surfactants, and pesticides, are not considered in this analysis as per the definition of NNC. Multiple ingredients in this pesticide formulation may exert strong antibacterial effects [34].

**Supplementary References**

1. X. Zhang, G. Tang, Z. Zhou, H. Wang, X. Li et al., Fabrication of enzyme-responsive prodrug self-assembly based on fluazinam for reducing toxicity to aquatic organisms. J. Agric. Food Chem. **71**(34), 12678-12687 (2023). <https://doi.org/10.1021/acs.jafc.3c03762>
2. G. Tang, Z. Zhou, X. Zhang, Y. Liu, G. Yan et al., Fabrication of supramolecular self-assembly of the schiff base complex for improving bioavailability of aldehyde-containing plant essential oil. Chem. Eng. J. **471**(1), 144471 (2023). <https://doi.org/10.1016/j.cej.2023.144471>
3. Y. Tian, G. Tang, Y. Li, Z. Zhou, X. Chen et al., A simple preparation process for an efficient nano-formulation: Small molecule self-assembly based on spinosad and sulfamic acid. Green Chem. 23(13), 4882-4891 (2021). <https://doi.org/10.1039/d1gc00971k>
4. W. N. Wu, M. H. Wan, Q. Fei, Y. Tian, S. J. Song et al., PDA@Ti(3)C(2)T(x) as a novel carrier for pesticide delivery and its application in plant protection: NIR-responsive controlled release and sustained antipest activity. Pest Manage. Sci. **77**(11), 4960-4970 (2021). <https://doi.org:10.1002/ps.6538>
5. W. L. Liang, A. X. Yu, G. D. Wang, F. Zheng, P. T. Hu et al., A novel water-based chitosan-La pesticide nanocarrier enhancing defense responses in rice (*Oryza sativa* L) growth. Carbohydr. Polym. **199**(1), 437-444 (2018). <https://doi.org:10.1016/j.carbpol.2018.07.042>
6. Z. Cao, X. Z. Ma, A. H. Zou, Z. X. Shi, S. Y. Xiang et al., Chitin nanocrystals supported copper: a new nanomaterial with high activity with *P. syringae* pv. *Tabaci*. Pest Manage. Sci. **79**(6), 2017-2028 (2023). <https://doi.org:10.1002/ps.7377>
7. Z. C. Wang, W. J. Xu, Z. Y. Meng, T. L. Fan, C. M. Yang et al., Development of spirotetramat nanoparticles based on mesoporous silica: improving the uptake and translocation of spirotetramat in plants. Environ. Sci. Pollut. Res. Int. **30**, 12618-12627 (2023). <https://doi.org:10.1007/s11356-022-23030-8>
8. L. T. Wu, H. Pan, W. L. Huang, M. J. Wang, Z. X. Hu et al., Self-assembled degradable iron-doped mesoporous silica nanoparticles for the smart delivery of prochloraz to improve plant protection and reduce environmental impact. Environ. Technol. Innovation **28**, 102890 (2022). <https://doi.org:10.1016/j.eti.2022.102890>
9. L. D. Cao, H. R. Zhang, Z. L. Zhou, C. L. Xu, Y. P. Shan et al., Fluorophore-free luminescent double-shelled hollow mesoporous silica nanoparticles as pesticide delivery vehicles. Nanoscale **10**, 20354-20365 (2018). <https://doi.org:10.1039/c8nr04626c>
10. X. M. Zhong, H. J. Wen, R. X. Zeng, H. K. Deng, G. F. Su et al., Zein-functionalized mesoporous silica as nanocarriers for nanopesticides with pH/enzyme dual responsive properties. Ind. Crops Prod. **188**(15), 115716 (2022). <https://doi.org:10.1016/j.indcrop.2022.115716>
11. H. Zhang, H. Qin, L. X. Li, X. T. Zhou, W. Wang et al., Preparation and Characterization of Controlled-Release Avermectin/Castor Oil-Based Polyurethane Nanoemulsions. J. Agric. Food Chem. **66**(26), 6552-6560 (2018). <https://doi.org:10.1021/acs.jafc.7b01401>
12. B. X. Liu, Y. Wang, F. Yang, H. X. Cui, D. C. Wu, Development of a Chlorantraniliprole Microcapsule Formulation with a High Loading Content and Controlled-Release Property. J. Agric. Food Chem. **66**(26), 6561-6568 (2018). <https://doi.org:10.1021/acs.jafc.7b01295>
13. X. L. Deng, P. Y. Zhao, X. M. Zhou, L. Y. Bai, Excellent sustained-release efficacy of herbicide quinclorac with cationic covalent organic frameworks. Chem. Eng. J. **405**(1), 126979 (2021). <https://doi.org:10.1016/j.cej.2020.126979>
14. Y. Liang, S. J. Wang, H. Q. Dong, S. W. Yu, H. J. Jia et al., Zeolitic Imidazole Framework-90-Based Pesticide Smart-Delivery System with Enhanced Antimicrobial Performance. Nanomaterials **12**(20), 3622 (2022). <https://doi.org:10.3390/nano12203622>
15. L. R. Ren, J. N. Zhao, W. J. Li, Q. J. Li, D. Q. Zhang et al., Site-Specific Controlled-Release Imidazolate Framework-8 for Dazomet Smart Delivery to Improve the Effective Utilization Rate and Reduce Biotoxicity. J. Agric. Food Chem. **70**(20), 5993-6005 (2022). <https://doi.org:10.1021/acs.jafc.2c00353>
16. Y. Liang, S. J. Wang, H. J. Jia, Y. J. Yao, J. H. Song et al., pH/redox/α-amylase triple responsive metal-organic framework composites for pest management and plant growth promotion. Microporous Mesoporous Mater. **344**, 112230 (2022). <https://doi.org:10.1016/j.micromeso.2022.112230>
17. Y. Y. Tian, Y. Q. Huang, X. H. Zhang, G. Tang, Y. H. Gao et al., Self-Assembled Nanoparticles of a Prodrug Conjugate Based on Pyrimethanil for Efficient Plant Disease Management. J. Agric. Food Chem. **70**(38), 11901-11910 (2022). <https://doi.org:10.1021/acs.jafc.2c04489>
18. Y. Y. Tian, X. H. Zhang, Y. Q. Huang, G. Tang, Y. H. Gao et al., Amphiphilic prodrug nano-micelles of fipronil coupled with natural carboxylic acids for improving physicochemical properties and reducing the toxicities to aquatic organisms. Chem. Eng. J. **439**(1), 135717 (2022). <https://doi.org:10.1016/j.cej.2022.135717>
19. K. L. Wei, Z. L. Li, Z. R. Zheng, Y. X. Gao, Q. L. Huang et al., Natural Glycyrrhizic Acid-Tailored Nanoparticles toward the Enhancement of Pesticide Bioavailability. Adv. Funct. Mater. Just Published, 2315493 (2024). <https://doi.org/10.1002/adfm.202315493>
20. Q. T. Ji, X. F. Mu, D. K. Hu, L. J. Fan, S. Z. Xiang et al., Fabrication of host-guest complexes between adamantane-functionalized 1,3,4-oxadiazoles and beta-cyclodextrin with improved control efficiency against intractable plant bacterial diseases. ACS Appl. Mater. Interfaces **14**(2), 2564-2577 (2022). <https://doi.org/10.1021/acsami.1c19758>
21. J. Yang, H. J. Ye, H. M. Xiang, X. Zhou, P. Y. Wang et al., Photo‐stimuli smart supramolecular self‐assembly of azobenzene/β‐cyclodextrin inclusion complex for controlling plant bacterial diseases. Adv. Funct. Mater. **33**(42), 2303206 (2023). <https://doi.org/10.1002/adfm.202303206>
22. G. Tang, Y. Tian, J. Niu, J. Tang, J. Yang et al., Development of carrier-free self-assembled nanoparticles based on fenhexamid and polyhexamethylene biguanide for sustainable plant disease management. Green Chem. **23**(6), 2531-2540 (2021). <https://doi.org/10.1039/d1gc00006c>
23. H. B. Qu, S. G. Wu, J. B. Gong A, sustainable and smart fungicide release platform through cocrystal nanocapsules for improved utilization rate and environmental safety. Chem. Eng. J. **473**(1), 145284 (2023). <https://doi.org/10.1016/j.cej.2023.145284>
24. R. S. Hazra, J. Roy, L. Jiang, D. C. Webster, Md. M. Rahman et al., Biobased, Macro-, and Nanoscale Fungicide Delivery Approaches for Plant Fungi Control. ACS Appl. Bio Mater. **6**(7), 2698–2711 (2023). <https://doi.org/10.1021/acsabm.3c00171>
25. L. P. Yang, A. E. Kaziem, Y. G. Lin, C. Li and Y. T. Tan, Carboxylated β-cyclodextrin anchored hollow mesoporous silica enhances insecticidal activity and reduces the toxicity of indoxacarb. Carbohydr. Polym. **266**(15), 118150 (2021). <https://doi.org/10.1016/j.carbpol.2021.118150>
26. J. T. Dong, W. Chen, J. G. Feng, X. Q. Liu, Y. Xu et al., Facile, Smart, and Degradable Metal–Organic Framework Nanopesticides Gated with FeIII-Tannic Acid Networks in Response to Seven Biological and Environmental Stimuli. ACS Appl. Mater. Interfaces **13**(16), 19507–19520 (2021). <https://doi.org/10.1021/acsami.1c04118>
27. Y. J. Ma, R. Zhan, H. Y. Shang, S. Zhen, L. J. Li et al., pH-Responsive ZIF-8-Based Metal–Organic-Framework Nanoparticles for Termite Control. ACS Appl. Nano Mater. **5**(8), 11864–11875 (2022). <https://doi.org/10.1021/acsanm.2c02856>
28. Y. Shen, C. C. An, J. J. Jiang, B. N. Huang, N. J. Li et al., Temperature-Dependent Nanogel for Pesticide Smart Delivery with Improved Foliar Dispersion and Bioactivity for Efficient Control of Multiple Pests. ACS Nano **16**(12), 20622–20632 (2022). <https://doi.org/10.1021/acsnano.2c07517>
29. W. L. Liang, Z. G. Xie, J. L. Cheng, D. X. Xiao, Q. Y. Xiong et al., A Light-Triggered pH-Responsive Metal–Organic Framework for Smart Delivery of Fungicide to Control Sclerotinia Diseases of Oilseed Rape. ACS Nano **15**(4), 6987–6997 (2021). <https://doi.org/10.1021/acsnano.0c10877>
30. P. T. Hu, L. Zhu, F. Zheng, J. Y. Lai, H. H. Xu et al., Graphene oxide as a pesticide carrier for enhancing fungicide activity against Magnaporthe oryzae. New J. Chem. **45**, 2649 (2021). <https://doi.org/10.1039/d0nj04721j>
31. M. Kah, R. S. Kookana, A. Gogos, T. D. Bucheli, A critical evaluation of nanopesticides and nanofertilizers against their conventional analogues. Nat. Nanotechnol. **13**(8), 677-684 (2018). <https://doi.org/10.1038/s41565-018-0131-1>
32. D. Wang, N. B. Saleh, A. Byro, R. Zepp, E. Sahle-Demessie et al., Nano-enabled pesticides for sustainable agriculture and global food security. Nat. Nanotechnol. **17**(4), 347-360 (2022). <https://doi.org/10.1038/s41565-022-01082-8>
33. E. Albalghiti, L. M. Stabryla, L. M. Gilbertson, J. B. Zimmerman, Towards resolution of antibacterial mechanisms in metal and metal oxide nanomaterials: a meta-analysis of the infuence of study design on mechanistic conclusions. Environ. Sci. Nano **8**, 37-66 (2020). <https://doi.org/10.1039/D0EN00949K>
34. M. Bae, A. Lewis, S. Liu, Y. Arcot, Y. T. Lin et al., Novel Biopesticides Based on Nanoencapsulation of Azadirachtin with Whey Protein to Control Fall Armyworm. J. Agric. Food Chem. **70**(26), 7900-7910 (2022). <https://doi.org/10.1021/acs.jafc.2c01558>
